# Sensor Fault Detection and Reliable Control of Singular Stochastic Systems with Time-Varying Delays

**DOI:** 10.3390/s25154667

**Published:** 2025-07-28

**Authors:** Yunling Shi, Haosen Yang, Gang Liu, Xiaolin He, Jajun Wang

**Affiliations:** 1 School of Automation, Harbin University of Science and Technology, Harbin 150000, China; 15650118560@163.com (H.Y.); 2420500009@stu.hrbust.edu.cn (X.H.); 15546412812@163.com (J.W.); 2 School of Electrical and Information Engineering, HeilongJiang University of Technology, Jixi 158100, China; liu.gang.1980@gmail.com

**Keywords:** singular nonlinear system, uncertain system, fault detection filter, time-varying delay, Markov jump system

## Abstract

In unmanned systems, especially in large-scale and complex ones, sensor and communication failures occur from time to time and are hard to avoid. Therefore, this paper studies the fault detection problem of a class of unknown nonlinear singular uncertain time-varying delay Markov jump systems (UNSUTVDMJSs). Firstly, the corresponding sliding mode controller (SMC) is designed by using the equivalent control principle, and the unknown nonlinearity is equivalently replaced by changing the system input. Then, a fault detection filter adapted to this system is designed, thereby obtaining the unknown nonlinear stochastic singular uncertain Augmented filter residual system (UNSSUAFRS) model. To obtain the sufficient conditions for the random admissibility of this augmented system, a weak infinitesimal generator was used to design the required Lyapunov-Krasovskii functional. With the help of the Lyapunov principle and $H_{\infty }$ performance analysis method, the sufficient conditions for the random admissibility of UNSSUAFRS under the $H_{\infty }$ performance index γ were derived. Finally, with the aid of the designed residual evaluation function and threshold, simulation analysis was conducted on the examples of DC servo motors and numerical calculation examples to verify the effectiveness and practicability of this fault detection filter.

## 1. Introduction

Modern unmanned systems, such as unmanned aerial vehicles [1,2,3], unmanned ground vehicles [4,5], unmanned underwater vehicles [6,7] and other autonomous cluster systems, play an important role in many fields such as military reconnaissance, disaster rescue, logistics distribution [8,9], infrastructure inspection and precision agriculture, thanks to their advantages of flexible deployment and strong adaptability. However, the core prerequisite for its wide deployment and in-depth application lies in the security and reliability of its operation. Unmanned systems often perform tasks in complex, dynamic or even unstructured environments. Their core components such as sensors, actuators, power systems and communication links are highly prone to failure due to mechanical wear, environmental interference, cyber attacks or human operational errors. Once it occurs and is not detected and dealt with in time, it may lead to mission failure at the least, and at the worst, it may cause disastrous consequences, resulting in significant casualties and property losses. Therefore, in this paper, nonlinearity, singular uncertainty and Markov jump will be utilized to model complex unmanned systems. To better meet the requirements of the actual system, time delay and perturbation are considered in the control system, and then the problem of fault detection is studied. Singular systems, also known as singular systems, possess the endogeneity of physical constraints and the coupling of multi-scale dynamics. Therefore, they can directly describe the characteristics of variable coupling, coexistence of algebraic constraints and dynamic evolution in complex systems such as distributed coordination of smart grids [10,11], multimodal control of high-speed aircraft [12,13], dynamic optimization of biochemical reactions, and precise operation of flexible robotic arms [14,15]. It has been widely applied in the modeling of complex engineering systems. The performance boundaries of modern engineering systems are constantly being pushed to the limit. Classical linear control theories (such as PID and LQG) face fundamental limitations in terms of global stability, transient performance and robustness. When the system behavior cannot satisfy the superposition principle, nonlinear systems begin to play an important role, from the attitude adjustment of ultra-maneuverable fighter jets [16,17] to the precise operation of micro-surgical robots [18,19]. From the transient stability of the smart grid [20,21] to the concentration optimization of bioreactors, the core dynamics of these scenarios are all dominated by strong nonlinear effects. In unmanned systems, the parameters, structure or dynamics of the control system are often uncertain due to inertia, damping, environmental interference or structural changes, etc. The time-delay phenomenon is a common problem in actual systems, often leading to phase lag, spectral distortion, energy accumulation, signal aliasing and failure of fault detection in the system, thereby undermining the system performance. In order to better describe the actual system, this paper will study the singular uncertain nonlinear system with time-varying delays.

Markovian Jump Systems (MJS) are a type of dynamic systems dominated by random machine mode switching, whose parameters or structures can randomly switch among a finite number of modes based on the Markov chain. Therefore, it can accurately describe the problem of random system changes caused by environmental interference, component damage, malicious destruction and other situations. It has been applied in aspects such as financial and stock market prediction [22,23], network control systems [24,25], and biological gene regulation systems. Therefore, MJSs has been highly favored, and its fault detection problem [26,27] has become a research hotspot in the industry. A large number of researchers have published corresponding research results. However, no scholar has conducted research on the sensor fault detection problem of singular uncertain nonlinear Markov jump systems. This paper will study the sensor fault detection problem of this complex system. In order to improve the detection efficiency, in this paper, the corresponding fault detection filter will be designed based on the given system model. Then, this filter will be used to generate residual signals and conduct comparisons to determine whether a fault has occurred. The main contributions of this paper are: (1.) An integral-type synovial controller was designed, which solved the nonlinear problem in uncertain Markov singular systems; (2.) Design a filter suitable for sensor fault detection in singular uncertain nonlinear time-varying delay Markov jump systems; (3.) Sufficient conditions for the adperability of the residual system of unknown nonlinear stochastic singular uncertain augmented filtering are given.

The overall structure of this article is as follows: The first part presents the system model and lists the lemmas to be used in the article; The second part designs an integral synovial controller to handle unknown nonlinear terms; The third part designs a filter suitable for sensor fault detection in singular uncertain nonlinear time-varying delay Markov jump systems; The fourth part proves the sufficient conditions for the adperability of the residual system of unknown nonlinear stochastic singular uncertain augmented filtering under passive conditions; The fifth part presents sufficient conditions for the adperability of unknown nonlinear stochastic singular uncertain augmented filtering residual systems under active conditions; The sixth part is simulation analysis to verify the effectiveness of the proposed and designed fault detection filter.

## 2. Materials and Methods

In this paper, a probability space is described by the triplet (O,Γ,P), where O represents the sample space, with each element within it representing a basic event; Γ represents the collection of subsets within the sample space, representing the event domain; and the Γ serves as the domain on which the probability measure P is defined, assigning probabilities to all events within the event domain. The switching mode {r(t),t∈[0,+∞)} in the system is determined by a Markov chain within a finite set of states S={1,2,⋯,N}, with its transition rate matrix denoted by $\Theta \overset{\Delta }{\;=}\left\{\lambda_{ij}\right\},\left(i,j\in S\right)$.

The above transfer rate matrix is controlled by the following formula:

(1) $$P=\left\{r(t+\Delta t)=j|r(t)=i\right\}=\left\{\begin{array}{c} \lambda_{ij}\times \Delta t+o\left(\Delta t\right),i\neq j\\ 1+\lambda_{ii}\times \Delta t+o\left(\Delta t\right),i=j \end{array}\right.$$

where r(t) represents the random jump of the system between different working conditions, environments or failure modes; Δt>0 represents the time step, $\lim_{\Delta t\to 0}\frac{o(\Delta t)}{\Delta t}=0$; $\lambda_{ij}>0$ represents the conversion rate from *i* mode to *j* mode, and $\lambda_{ii}=-\sum_{j\neq i}^{N}\lambda_{ij}$ represents the conversion rate that remains unchanged while staying in the *i* mode.

Based on actual needs, this article considers the following UNSUTVDMJSs:

(2) $$\left\{\begin{array}{c} E\dot{x}\left(t\right)=\left(A\left(r\left(t\right)\right)+\Delta A\left(r\left(t\right)\right)\right)x\left(t\right)+\left(A_{d}\left(r\left(t\right)\right)+\Delta A_{d}\left(r\left(t\right)\right)\right)x\left(t-d\left(t\right)\right)\\ \;\;\;+B\left(r\left(t\right)\right)\left(u\left(t\right)+\Phi_{i}\left(x\left(t\right),t\right)\right)+F\left(r\left(t\right)\right)f\left(t\right)+H\left(r\left(t\right)\right)\omega \left(t\right)\\ y\left(t\right)=C\left(r\left(t\right)\right)x\left(t\right)+C_{d}\left(r\left(t\right)\right)x\left(t-d\left(t\right)\right)+D\left(r\left(t\right)\right)f\left(t\right)+G\left(r\left(t\right)\right)\omega \left(t\right)\\ z\left(t\right)=L\left(r\left(t\right)\right)x\left(t\right)+L_{d}\left(r\left(t\right)\right)x\left(t-d\left(t\right)\right)\\ x\left(t\right)=\chi \left(t\right),\;\forall t\in \left[-d,0\right) \end{array}\right.$$

For notational convenience, let r(t)=i∈S, the system matrix A(r(t)), ΔA(r(t)), $A_{d}\left(r\left(t\right)\right)$, $\Delta A_{d}\left(r\left(t\right)\right)$, B(r(t)), F(r(t)), H(r(t)), C(r(t)), $C_{d}\left(r\left(t\right)\right)$, D(r(t)), G(r(t)), L(r(t)), $L_{d}\left(r\left(t\right)\right)$ can be expressed as $A_{i}$, $A_{di}$, $B_{i}$, $C_{i}$, ${C_{d}}_{i}$, $D_{i}$, $F_{i}$, $G_{i}$, $H_{i}$, $L_{i}$, $L_{di}$, Then Formula (2) can be transformed into the following formula:

(3) $$\left\{\begin{array}{c} E\dot{x}\left(t\right)=\left(A_{i}+\Delta A_{i}\right))x\left(t\right)+\left(A_{di}+\Delta A_{di}\right)x\left(t-d\left(t\right)\right)+B_{i}\left(u\left(t\right)+\Phi_{i}\left(x\left(t\right),t\right)\right)\\ \;\;\;+F_{i}f\left(t\right)+H_{i}\omega \left(t\right)\\ y\left(t\right)=C_{i}x\left(t\right)+C_{di}x\left(t-d\left(t\right)\right)+D_{i}f\left(t\right)+G_{i}\omega \left(t\right)\\ z\left(t\right)=L_{i}x\left(t\right)+L_{di}x\left(t-d\left(t\right)\right)\\ x\left(t\right)=\chi \left(t\right),\;\forall t\in \left[-d,0\right] \end{array}\right.$$

where the state vector of the system is represented by $x\left(t\right)\in R^{n}$; $\dot{x}\left(t\right)$ is the derivative of x(t); $x\left(t-d\left(t\right)\right)\in R^{n}$ is introduced to describe the state vector affected by time-varying delays; The input vector is denoted by $u\left(t\right)\in R^{n}$; $y\left(t\right)\in R^{n}$ represents the output vector; The fault signal is expressed as $f\left(t\right)\in R^{f}$; $\omega \left(t\right)\in R^{\omega }$ is the system interference signal; $\Phi_{i}\left(x\left(t\right),t\right))$ represents an unknown nonlinear term, satisfied $∥\Phi_{i}\left(x\left(t\right),t\right)∥<\eta ∥x(t)∥$; $A_{i}$, $A_{di}$, $B_{i}$, $C_{i}$, ${C_{d}}_{i}$, $D_{i}$, $F_{i}$, $G_{i}$, $H_{i}$, $L_{i}$, $L_{di}$ is a known dimensional matrix; $\Delta A_{i}$, $\Delta A_{di}$ is an uncertain term. *E* is a singular matrix that satisfies rank(E)=r<n.

Definition 1 [28]. Stochastic admissibility conditions for singular Markov systems:

(1) Random regularized, If i∈S, $\det(sE-A_{i})\neq 0$, Then the system is said to be randomly regular;
(2) Random and pulse-free, If i∈S, $\deg\left(\det(sE-A_{i})\right)=rank\left(E\right)$, Then the system is said to be random and pulse-free;
(3) Stochastic stability, If for any initial state $x\left(0\right)\in R^{n}$, r(0)∈I, there exists a scale $M(x_{0},r_{0})>0$, in order for the subsequent inequality to be satisfied:
  (4) $$E\left\{\int_{0}^{\infty }{∥x(t)∥}^{2}|\left(x_{0},r_{0}\right)\right\}\leq M\left(x_{0},r_{0}\right)$$
(4) If the system is randomly regular, randomly pulse-free, and randomly stable, it is proved that the system is randomly admissible.

Definition 2 [29]. Given a scalar γ>0, under certain conditions, if the following two conditions are met, UNSSUAFRS (3) is said to be randomly permissible and has the $H_{\infty }$ performance metric γ.

(1) When φ(t)=0, t≥0 and when condition (3) of Definition 1 is satisfied, system is randomly stable.
(2) When φ(t)≠0, t≥0, it exists under zero initial conditions, the following inequality holds:

(5) $$E\left\{\int_{0}^{\infty }e^{T}\left(t\right)e\left(t\right)dt\right\}<\gamma^{2}E\left\{\int_{0}^{\infty }\varphi^{T}\left(t\right)\varphi \left(t\right)dt\right\}$$

Lemma 1 [30]. (based on Wirtinger’s integral inequality [31])

For any positive definite matrix $R\in R^{n\times n}>0$, given scalars *b* and *a*, which are the upper and lower bounds of the integral respectively, satisfy condition b>a. For all continuously differentiable functions ν(s), the following inequality holds in $\left[a,b\right]\to R^{n\times n}$:

(6) $$\int_{a}^{b}\nu^{T}\left(s\right)R\nu \left(s\right)ds\geq \frac{1}{b-a}\psi_{1}^{T}R\psi_{1}+\frac{3}{b-a}\psi_{2}^{T}R\psi_{2}$$

where $\psi_{1}=\int_{a}^{b}\nu \left(s\right)ds$, $\psi_{2}=\int_{a}^{b}\nu \left(s\right)ds-\frac{2}{b-a}\int_{a}^{b}\int_{s}^{b}\nu \left(u\right)duds$.

Lemma 2 [32]. For a symmetric positive definite matrix $\;R\;\in \;R^{n\times n}$, and for all differentiable functions $\dot{x}\left(s\right)$ satisfying $\left[a,b\right]\to R^{n}$, the following inequality is valid:

(7) $$\left(b-a\right)\int_{a}^{b}{\dot{x}}^{T}\left(s\right)R\dot{x}\left(s\right)ds\geq \theta_{1}^{T}\Omega \theta_{1}+3\theta_{2}^{T}\Omega \theta_{2}+5\theta_{3}^{T}\Omega \theta_{3}$$

where

$\left\{\begin{array}{c} \theta_{1}=x\left(b\right)-x\left(a\right)\\ \theta_{2}=x\left(b\right)+x\left(a\right)-\frac{2}{b-a}\int_{a}^{b}x\left(s\right)ds\\ \theta_{3}=x\left(b\right)-x\left(a\right)+\frac{6}{b-a}\int_{a}^{b}x\left(s\right)ds-\frac{12}{{\left(b-a\right)}^{2}}\int_{a}^{b}\int_{s}^{b}\omega \left(u\right)duds \end{array}\right.$

Lemma 3 [33].The inequality presented below is satisfied for all scalars ε>0, where $X_{i}$ and $Y_{i}$ are assumed to be real constant matrices.

(8) $$X_{i}^{T}Y_{i}+X_{i}Y_{i}^{T}\leq \varepsilon X_{i}^{T}X_{i}+\varepsilon^{-1}Y_{i}Y_{i}^{T}$$

Lemma 4 [31]. The structural uncertainties $\Delta A_{i}$ and $\Delta A_{di}$ are norm-bounded, that is:

(9) $$\left[\begin{array}{c} \Delta A_{i}\\ \Delta A_{di} \end{array}\right]=\left[\begin{array}{c} M_{1i}\\ M_{2i} \end{array}\right]F_{i}\left(t\right)N_{i}$$

Lemma 5 [34]. System (3) is randomly admissible if and only if there exists a set of positive definite matrices *P* such that:

(10) $$A_{i}^{T}P_{i}+P_{i}^{T}A_{i}+\sum_{j=1}^{N}\lambda_{ij}E^{T}P_{j}E<0$$

(11) $$E^{T}P_{i}=P_{i}^{T}E\geq 0$$

To achieve the purpose of fault detection, the following residual evaluation function J(r(t)) and threshold $J_{th}$ are set:

The residual evaluation function J(r(t)) and threshold $J_{th}$ selected in this chapter are as follows:

(12) $$\left\{\begin{array}{c} J\left(r\left(t\right)\right)={\left\{\int_{k_{0}}^{k_{0}+T}r^{T}\left(t\right)r\left(t\right)\right\}}^{\frac{1}{2}}\\ J_{th}=\underset{\varphi (t)\neq 0,u(t)\neq 0}{\sup}E\left[J(r(t))\right] \end{array}\right.$$

At this point, the fault detection strategy is:

(13) $$\left\{\begin{array}{cc} \begin{array}{c} J\left(r\left(t\right)\right)\leq J_{th}\\ J\left(r\left(t\right)\right)>J_{th} \end{array} & \begin{array}{c} No\;fault\\ fault \end{array} \end{array}\right.$$

## 3. Design the Sliding Mode Controller

To handle the unknown nonlinear term $M_{i},N_{i}\in R^{n}$, this section designs an integral sliding mode controller. By applying the equivalent control principle, the control information is input through the sliding mode controller, and the corresponding control strategy is adopted to make the system stable on this sliding mode surface. By constructing the Lyapunov function, the stability of system (3) under this sliding mode controller is further proved, thereby ensuring that the system state trajectory can converge to the switching surface within a finite time and maintain stable sliding mode dynamic characteristics.

Design control feedback $u\left(t\right)=K_{i}x\left(t\right)$. By adjusting the proportional gain $K_{i}$, the response speed and stability of the system can be changed. It can also assist in equivalent control. By applying the linearization compensation of the control system, the nonlinearity of the equivalent control system can be achieved, thereby optimizing the closed-loop dynamics.

First, set up the sliding mold surface s(t):

(14) $$\begin{array}{c} s\left(t\right)=T_{i}Ex\left(t\right)-\int_{0}^{t}T_{i}\left(A_{i}+B_{i}K_{i}\right)x\left(s\right)ds-\int_{0}^{t}T_{i}A_{di}x\left(s-d\left(s\right)\right)ds\\ \;\;-\int_{0}^{t}T_{i}\left(F_{i}f\left(s\right)+H_{i}\omega \left(s\right)\right)ds \end{array}$$

where design $T_{i}=B_{i}^{T}$, and it satisfies that $T_{i}B_{i}$ is an invertible matrix.

Integrating both sides of the equation of state in Formula (3) simultaneously over 0→t yields:

(15) $$\begin{array}{c} \int_{0}^{t}E\dot{x}\left(s\right)ds=\int_{0}^{t}\left(A_{i}+\Delta A_{i}\right))x\left(s\right)ds+\int_{0}^{t}\left(A_{di}+\Delta A_{di}\right)x\left(s-d\left(s\right)\right)ds\\ \;\;\;\;\;+\int_{0}^{t}B_{i}\left(u\left(s\right)+\Phi_{i}\left(x\left(s\right),s\right)\right)ds+\int_{0}^{t}T_{i}\left(F_{i}f\left(s\right)+H_{i}\omega \left(s\right)\right)ds \end{array}$$

Then, it is expected that the system will approach the sliding mode surface and remain stable within a finite time, that is s(t)=0, $\dot{s}\left(t\right)=0$. By differentiating the sliding mode surface s(t), we can obtain:

(16) $$\dot{s}\left(t\right)=T_{i}B_{i}\left(u\left(t\right)+\Phi_{i}\right)+T_{i}\Delta A_{i}x\left(t\right)-T_{i}B_{i}K_{i}x\left(t\right)+T_{i}\Delta A_{di}x\left(t-d\left(t\right)\right)$$

Combining Formulas (14)–(16), the equivalent control input $U_{eq}$ under sliding mode control can be obtained:

(17) $$U_{eq}=K_{i}x\left(t\right)-\Phi_{i}-{\left(T_{i}B_{i}\right)}^{-1}T_{i}\Delta A_{i}x\left(t\right)-{\left(T_{i}B_{i}\right)}^{-1}T_{i}\Delta A_{di}x\left(t-d\left(t\right)\right)$$

**Remark** **1.**  *To eliminate the high-frequency buffeting caused by the equivalent control input*  $U_{eq}$*, a proportional term*  $K_{i}x\left(t\right)$ *is added to the sliding film control strategy. This is because before the state trajectory reaches the sliding mode surface, the proportional feedback*  $K_{i}x\left(t\right)$ *can accelerate the convergence speed of the state*  x(t)*, thereby speeding up the time for the state trajectory to reach the sliding mode surface*  s→0*, reducing the system’s reliance on the switching term, and ultimately lowering the buffeting amplitude*.

**Theorem** **1.**  *For the nonlinear problem of system (3), combined with the sliding mode control strategy in (17), a sliding mode controller adapted to this system was designed, enabling the nonlinear term $\Phi_{i}\left(x\left(t\right),t\right)$ trajectory in the system to reach s(t)=0 within a finite time. The sliding mode control strategy is as follows:*

(18) $$u\left(t\right)=K_{i}x\left(t\right)-{\left(T_{i}B_{i}\right)}^{-1}\lambda_{i}\left(t\right)\mathrm{sgn}\left(s\left(t\right)\right)$$

*where $\lambda_{i}\left(t\right)=h+\left(∥T_{i}M_{1i}∥∥N_{i}∥+∥T_{i}B_{i}\eta ∥\right)∥x(t)∥+∥T_{i}M_{2i}∥∥N_{i}∥∥x(t-d(t))∥$*

**Proof.**  The Lyapunov function is designed as follows:

(19) $$v\left(t\right)=\frac{1}{2}s^{T}\left(t\right)s\left(t\right)$$

By differentiating Formula (19) and combining it with Formula (16), we can obtain:

(20) $$\begin{array}{c} \dot{V}\left(t\right)=s^{T}\left(t\right)\dot{s}\left(t\right)\\ \leq ∥s(t)∥\left(∥T_{i}M_{1i}∥∥N_{i}∥∥x(t)∥+∥T_{i}M_{2i}∥∥N_{i}∥∥x(t-d(t))∥\right)\\ +s^{T}\left(t\right)T_{i}B_{i}\left(u\left(t\right)+\Phi_{i}\right)-s^{T}\left(t\right)T_{i}B_{i}K_{i}x\left(t\right) \end{array}$$

Combining (18) and (21), it can be inferred that:

(21) $$\dot{V}\left(t\right)\leq -h∥s(t)∥$$

where the scalar h>0.

**Remark** **2.**  *From the above equation, it can be easily seen that*  $\dot{V}\left(t\right)$ *is always negative, and its state trajectory can reach the preset sliding mode surface within a finite time. This indicates that the designed sliding mode controller can stabilize UNSSUAFRS (3). Therefore, the proof of Theorem 1 is completed.* □

After being optimized by this sliding mode controller, System (3) can become:

(22) $$\begin{array}{c} E\dot{x}\left(t\right)=\left(A_{i}+B_{Ti}\Delta A_{i}\right)x\left(t\right)+\left(A_{di}+B_{Ti}\Delta A_{di}\right)x\left(t-d\left(t\right)\right)+B_{i}u\left(t\right)\\ +F_{i}f\left(t\right)+H_{i}\omega \left(t\right) \end{array}$$

where $B_{Ti}=I-B_{i}{\left(T_{i}B_{i}\right)}^{T}T_{i}$

## 4. Design a Fault Detection Filter for UNSUTVDMJSs

To facilitate the estimation of z(t), a full-order mode-dependent filtering scheme is adopted:

(23) $$\left\{\begin{array}{c} {\dot{x}}_{f}\left(t\right)=A_{f}\left(r\left(t\right)\right)x_{f}\left(t\right)+B_{f}\left(r\left(t\right)\right)y\left(t\right)\\ z_{f}\left(t\right)=C_{f}\left(r\left(t\right)\right)x_{f}\left(t\right)+D_{f}\left(r\left(t\right)\right)y\left(t\right) \end{array}\right.$$

where the notation $x_{f}\left(t\right)\in R^{n}$ is adopted to describe the state vector associated with the filter and $z_{f}\left(t\right)\in R^{n}$ represents the estimated vector of the filter.

The initial condition of this fault detection filter system is $x_{f}\left(0\right)=x_{f0}$, and define $\hat{x}\left(t\right)={\left[\begin{array}{cc} x^{T}\left(t\right) & {x_{f}}^{T}\left(t\right) \end{array}\right]}^{T}$, $\varphi \left(t\right)=\left[\begin{array}{ccc} u(t) & f(t) & \omega (t) \end{array}\right]$, $r\left(t\right)=\hat{z}\left(t\right)=z\left(t\right)-z_{f}\left(t\right)$.

By combining the system (22) and the filter (23), the following UNSSUAFRS were obtained:

(24) E¯x^˙(t)=(A¯i+ΔA¯i))x^(t)+(A¯di+ΔA¯di)x^(t−d(t))+Γ1iφ(t)r(t)=L¯ix^(t)+L¯dix^(t−d(t))+Γ2iφ(t)

where $\bar{E}=\left[\begin{array}{cc} E & 0\\ 0 & I \end{array}\right]$, ${\bar{A}}_{i}=\left[\begin{array}{cc} A_{i} & 0\\ B_{fi}C_{i} & A_{fi} \end{array}\right]$, $\Delta {\bar{A}}_{i}=\left[\begin{array}{cc} B_{Ti}\Delta A_{i} & 0\\ 0 & 0 \end{array}\right]$, ${\bar{A}}_{di}=\left[\begin{array}{cc} A_{di} & 0\\ B_{fi}C_{di} & 0 \end{array}\right]$, $\Delta {\bar{A}}_{di}=\left[\begin{array}{cc} B_{Ti}\Delta A_{di} & 0\\ 0 & 0 \end{array}\right]$, $\Gamma_{1i}=\left[\begin{array}{ccc} B_{i} & F_{i} & H_{i}\\ 0 & B_{fi}D_{i} & B_{fi}G_{i} \end{array}\right]$, $\Gamma_{2i}=\left[\begin{array}{ccc} 0 & D_{fi}D_{i} & D_{fi}G_{i} \end{array}\right]$, ${\bar{L}}_{i}=\left[\begin{array}{cc} L_{i}-D_{f}C_{i} & C_{fi} \end{array}\right]$, ${\bar{L}}_{di}=\left[\begin{array}{cc} L_{di}-D_{f}C_{di} & 0 \end{array}\right]$.

**Remark** **3.**  *In fact, UNSSUAFRS (24) possesses the characteristics of a singular system. To better conduct in-depth research and analysis on the admissibility of the UNSSUAFRS system (24), it is essential to ensure that the solutions obtained by UNSSUAFRS (24) are valid when input*  u(t)=0.

At this point, UNSSUAFRS (24) under passive conditions is:

(25) E¯x^˙(t)=(A¯i+ΔA¯i))x^(t)+(A¯di+ΔA¯di)x^(t−d(t))x(t)=χ(t), ∀t∈[−d,0]

The main purpose of this paper is to prove the random admisability of UNSSUAFRS (24) under active conditions, and on this basis, obtain the effective parameters of its full-order fault detection filter (23) and meet its corresponding performance indicators. To achieve the above results, it is necessary to first prove the random admisability of the system (25) under passive conditions.

## 5. Random Admissibility Analysis of UNSSUAFRS Under Passive Conditions

In this section, by means of the Lyapunov principle and $H_{\infty }$ performance analysis method, the sufficient conditions for random admissibility to satisfy the UNSSUAFRS (25) $H_{\infty }$ performance index γ under passive conditions are derived.

**Theorem** **2.**  *Given a scalar η>0, if there exists a symmetric positive definite matrix $P_{i}>0$, $Q_{1}>0$, $Q_{2}>0$, $R_{1}>0$, $R_{2}>0$ and an arbitrary column full-rank matrix S such that ${\bar{E}}^{T}\bar{S}=0$ holds the LMI applicable to any i,j∈I, then UNSSUAFRS (25) under passive conditions is randomly admistive.*

(26) $$\begin{array}{c} P_{i}={P_{i}}^{T}=\left[\begin{array}{cc} P_{11i} & P_{12i}\\ {}* & P_{22i} \end{array}\right]>0,Q_{1}=Q_{1}^{T}=\left[\begin{array}{cc} Q_{11} & Q_{12}\\ {}* & Q_{13} \end{array}\right]>0,\\ Q_{2}=Q_{2}^{T}=\left[\begin{array}{cc} Q_{21} & Q_{22}\\ {}* & Q_{23} \end{array}\right]>0,R_{1}={R_{1}}^{T}=\left[\begin{array}{cc} R_{11} & R_{12}\\ {}* & R_{13} \end{array}\right]>0,\\ \begin{array}{cc} R_{2}={R_{2}}^{T}=\left[\begin{array}{cc} R_{21} & R_{22}\\ {}* & R_{23} \end{array}\right]>0, & \bar{S}=\left[\begin{array}{c} 0\\ S \end{array}\right] \end{array} \end{array}$$

(27) $$\tilde{\Xi }=\left[\begin{array}{ccccc} \Omega_{11} & \Omega_{12} & 3{\bar{E}}^{T}R_{2}\bar{E} & -24{\bar{E}}^{T}R_{2}\bar{E} & 60{\bar{E}}^{T}R_{2}\bar{E}\\ {}* & \Omega_{22} & 0 & 0 & 0\\ {}* & * & \Omega_{33} & 36{\bar{E}}^{T}R_{2}\bar{E} & -60{\bar{E}}^{T}R_{2}\bar{E}\\ {}* & * & * & -4d^{2}R_{1}-192{\bar{E}}^{T}R_{2}\bar{E} & 6d^{2}R_{1}+360{\bar{E}}^{T}R_{2}\bar{E}\\ {}* & * & * & * & -12d^{2}R_{1}-720{\bar{E}}^{T}R_{2}\bar{E} \end{array}\right]<0$$

where

(28) $$\begin{array}{c} \Omega_{11}=sym\left\{{\bar{A}}_{i}^{T}\left(P_{i}\bar{E}+\bar{S}{\bar{R}}_{i}^{T}\right)\right\}+Q_{1}+Q_{2}+d^{2}R_{1}+d^{2}{\bar{A}}_{i}^{T}R_{2}{\bar{A}}_{i}\\ -9{\bar{E}}^{T}R_{2}\bar{E}+\sum_{j=1}^{N}\lambda_{ij}{\bar{E}}^{T}P_{j}\bar{E}\\ \Omega_{12}=\left(P_{i}\bar{E}+\bar{S}{\bar{R}}_{i}^{T}\right){\bar{A}}_{di}+d^{2}{\bar{A}}_{di}^{T}R_{2}{\bar{A}}_{di}\\ \Omega_{22}=-\left(1-\mu \right)Q_{2}\\ \Omega_{33}=-Q_{1}-9{\bar{E}}^{T}R_{2}\bar{E} \end{array}$$

**Proof.**  As defined in Definition 1, to ensure the random admissibility of UNSSUAFRS (25) under passive conditions, it is necessary to first prove its random regularity and random pulse-free nature.

Suppose there exists an invertible matrix $M_{i}$ and $N_{i}$ it satisfies the following conditions:

(1) For the singular matrix *E* of the system, the following formula is established:
  (29) $$E=M_{i}^{-1}\left[\begin{array}{cc} I & 0\\ 0 & 0 \end{array}\right]N_{i}^{-1}$$
(2) For the parameter matrix $A_{i}$ of the system, the following formula is established:
  (30) $$A_{i}=M_{i}^{-1}\left[\begin{array}{cc} A_{11i} & A_{12i}\\ A_{21i} & A_{22i} \end{array}\right]N_{i}^{-1}$$
(3) If $A_{4i}$ is reversible, then the system is randomly regular and randomly pulse-free.

A non-singular matrix that satisfies the following conditions is given:

(31) $$M_{i}^{-T}P_{i}M_{i}^{-1}=\left[\begin{array}{cc} P_{11i} & P_{12i}\\ {}* & P_{22i} \end{array}\right]$$

Substituting Formulas (29) and (31) into Formula (11), the following equation is obtained:

(32) $$\begin{array}{c} E^{T}P_{i}=P_{i}^{T}E\\ =N_{i}^{-T}\left[\begin{array}{cc} I & 0\\ 0 & 0 \end{array}\right]M_{i}^{-T}M_{i}^{T}\left[\begin{array}{cc} P_{11i} & P_{12i}\\ {}* & P_{22i} \end{array}\right]N_{i}^{-1}\\ =N_{i}^{-T}\left[\begin{array}{cc} P_{11i} & P_{12i}\\ {}* & P_{22i} \end{array}\right]M_{i}M_{i}^{-T}\left[\begin{array}{cc} I & 0\\ 0 & 0 \end{array}\right]N_{i}^{-1} \end{array}$$

Multiplying both sides of Formula (32) in Lemma 5 by $N^{T}$ and *N* respectively, and combining Formulas (29)–(31), Formula (10) can be transformed into the following inequality:

(33) $$\left[\begin{array}{cc} {}* & *\\ {}* & A_{22i}^{T}P_{22i}+P_{22i}^{T}A_{22i} \end{array}\right]<0$$

where “∗” represents a block matrix. The matrix block ∗ does not affect the overall structure of the inequality or the study of this problem, so it is ignored in this paper.

If Formula (33) holds, we can obtain:

(34) $$A_{4i}^{T}P_{4i}+P_{4i}^{T}A_{4i}<0$$

Once Formula (34) holds, $A_{4i}$ is invertible. According to Lemma 5, when Formulas (10) and (11) hold, system (25) possesses random regularity and random pulseless property.

To effectively handle the conservation problem of time-varying delays, the Wirtinger inequality was introduced, and the corresponding modal-dependent Lyapunov function V(t) was established. Combined with the coupling term $\sum_{j=1}^{N}\lambda_{ij}{\bar{E}}^{T}P_{j}\bar{E}$, the stability of the system (25) under random switching was guaranteed:

(35) $$V\left(t\right)=V_{1}\left(t\right)+V_{2}\left(t\right)+V_{3}\left(t\right)+V_{4}\left(t\right)$$

where

(36) V1(t)=xT(t)E¯TPiE¯x(t)V2(t)=∫t−dtx^T(s)Q1x^(s)ds+∫t−d(t)tx^T(s)Q2x^(s)dsV3(t)=d∫−d0∫t+θtx^T(s)R1x^(s)dsdθV4(t)=d∫−d0∫t+θtx^˙T(s)E¯TR2E¯x^˙(s)dsdθ

Let δV(t) be a weak infinitesimal of a random process, where δ is used to represent the weak infinitesimal operator of V(t), that is

(37) $$\begin{array}{c} \delta V\left(t\right)=\lim_{\Delta t\to 0}\frac{1}{\Delta t}\left\{E\left[V\left(\hat{x}\left(t+\Delta t\right)\right),r_{t+\Delta t}|\hat{x}\left(t\right),r_{t}=i\right]-V\left(\hat{x}\left(t\right)\right)\right\}\\ =\delta V_{1}\left(t\right)+\delta V_{2}\left(t\right)+\delta V_{3}\left(t\right)+\delta V_{4}\left(t\right) \end{array}$$

where

(38) δV1(t)=x^˙T(t)E¯TPiE¯x^(t)+x^T(t)E¯TPiE¯x^˙(t)+x^T(t)∑j=1NλijE¯TPjE¯x^(t)=(A¯i+ΔA¯i)x^(t)+(A¯di+ΔA¯di)x^(t−d(t))TPiE¯x^(t)+x^T(t)E¯TPi(A¯i+ΔA¯)x^(t)+(A¯di+ΔA¯di)x^(t−d(t))+x^T(t)∑j=1NλijE¯TPjE¯x^(t)

(39) $$\begin{array}{c} \delta V_{2}\left(t\right)\leq {\hat{x}}^{T}\left(t\right)\left(Q_{1}+Q_{2}\right)\hat{x}\left(t\right)-{\hat{x}}^{T}\left(t-d\right)Q_{1}\hat{x}\left(t-d\right)\\ -\left(1-u\right){\hat{x}}^{T}\left(t-d\left(t\right)\right)Q_{2}\hat{x}\left(t-d\left(t\right)\right) \end{array}$$

(40) $$\delta V_{3}\left(t\right)=d^{2}{\hat{x}}^{T}\left(t\right)R_{1}\hat{x}\left(t\right)-d\int_{t-d}^{t}{\hat{x}}^{T}\left(s\right)R_{1}\hat{x}\left(s\right)ds$$

(41) δV4(t)=d2x^˙(t)E¯TR2E¯x^˙(t)−d∫t−dtx^˙T(s)E¯TR2E¯x^˙(s)ds=d2(A¯i+ΔA¯i)x^(t)+(A¯di+ΔA¯di)x^(t−d(t))TR2(A¯i+ΔA¯i)x^(t)+(A¯di+ΔA¯di)x^(t−d(t))−d∫t−dtx^˙T(s)E¯TR2E¯x^˙(s)ds

According to Lemma 1, by scaling the cross-integration term in (40), we can obtain:

(42) $$-d\int_{t-d}^{t}{\hat{x}}^{T}\left(s\right)R_{1}\hat{x}\left(s\right)ds<{\left(\int_{t-d}^{t}\hat{x}\left(s\right)ds\right)}^{T}R_{1}\left(\int_{t-d}^{t}\hat{x}\left(s\right)ds\right)+3\psi_{2}^{T}R\psi_{2}$$

where $\psi_{2}=\int_{t-d}^{t}\hat{x}\left(s\right)ds-\frac{2}{d}\int_{t-d}^{t}\int_{s}^{t}\hat{x}\left(u\right)duds$.

According to Lemma 2, by scaling the cross-integration term in (41), we can obtain:

(43) −d∫t−dtx^˙T(s)E¯TR2E¯x^˙(s)ds<x^t−x^t−dTR2x^t−x^t−d+3θ2TR2θ2+5θ3TR2θ3

where

$\begin{array}{c} \theta_{2}=\hat{x}\left(t\right)+\hat{x}\left(t-d\right)-\frac{2}{d}\int_{t-d}^{t}\hat{x}\left(s\right)ds\\ \theta_{3}=\hat{x}\left(t\right)-\hat{x}\left(t-d\right)+\frac{6}{d}\int_{t-d}^{t}\hat{x}\left(s\right)ds-\frac{12}{d^{2}}\int_{t-d}^{t}\int_{s}^{t}\hat{x}\left(u\right)duds \end{array}$

When ${\bar{E}}^{T}\bar{S}=0$, the following zero equation applicable to any matrix can be derived:

(44) Λx^(t)=x^˙(t)E¯TS¯R¯iTx^(t)+x^(t)R¯iS¯TE¯x^˙(t)=A¯ix^(t)+A¯dix^(t−d(t))TS¯R¯iTx^(t)+x^(t)R¯iS¯TA¯ix^(t)+A¯dix^(t−d(t))=0

Add Equations (39) to (44) to obtain the weak infinitesimal operator δV(t):

(45) $$\begin{array}{c} \delta V\left(t\right)\leq {\left[{\bar{A}}_{i}\hat{x}\left(t\right)+{\bar{A}}_{di}\hat{x}\left(t-d\left(t\right)\right)\right]}^{T}P_{i}\bar{E}\hat{x}\left(t\right)\\ +{\hat{x}}^{T}\left(t\right){\bar{E}}^{T}P_{i}\left[{\bar{A}}_{i}\hat{x}\left(t\right)+{\bar{A}}_{di}\hat{x}\left(t-d\left(t\right)\right)\right]\\ +{\hat{x}}^{T}\left(t\right)\sum_{j=1}^{N}\lambda_{ij}{\bar{E}}^{T}P_{j}\bar{E}\hat{x}\left(t\right)+{\hat{x}}^{T}\left(t\right)\left(Q_{1}+Q_{2}\right)\hat{x}\left(t\right)\\ -{\hat{x}}^{T}\left(t-d\right)Q_{1}\hat{x}\left(t-d\right)-\left(1-\mu \right){\hat{x}}^{T}\left(t-d\left(t\right)\right)Q_{2}\hat{x}\left(t-d\left(t\right)\right)\\ -9{\hat{x}}^{T}\left(t\right){\bar{E}}^{T}R_{2}\bar{E}\hat{x}\left(t\right)-24{\hat{x}}^{T}\left(t\right){\bar{E}}^{T}R_{2}\bar{E}\int_{t-d}^{t}\hat{x}\left(s\right)ds\\ +{\hat{x}}^{T}\left(t-d\right)\left(36{\bar{E}}^{T}R_{2}\bar{E}\right)\int_{t-d}^{t}\hat{x}\left(s\right)ds\\ +{\hat{x}}^{T}\left(t\right)\left(60{\bar{E}}^{T}R_{2}\bar{E}\right)\int_{t-d}^{t}\int_{\theta }^{t}\hat{x}\left(s\right)dsd\theta \\ +{\hat{x}}^{T}\left(t\right)\left(-60{\bar{E}}^{T}R_{2}\bar{E}\right)\int_{t-d}^{t}\int_{\theta }^{t}\hat{x}\left(s\right)dsd\theta +d{\hat{x}}^{T}\left(t\right)R_{1}\hat{x}\left(t\right)\\ -\int_{t-d}^{t}{\hat{x}}^{T}\left(s\right)ds\left(-4d^{2}R_{1}-192{\bar{E}}^{T}R_{2}\bar{E}\right)\int_{t-d}^{t}\hat{x}\left(s\right)ds\\ +\int_{t-d}^{t}{\hat{x}}^{T}\left(s\right)ds\left(6d^{2}R_{1}+360{\bar{E}}^{T}R_{2}\bar{E}\right)\int_{t-d}^{t}\int_{\theta }^{t}{\hat{x}}^{T}\left(s\right)dsd\theta \\ +\int_{t-d}^{t}\int_{\theta }^{t}{\hat{x}}^{T}\left(s\right)dsd\theta \left(-12d^{2}R_{1}-720{\bar{E}}^{T}R_{2}\bar{E}\right)\int_{t-d}^{t}\int_{\theta }^{t}\hat{x}\left(s\right)dsd\theta \\ +d^{2}{\left[({\bar{A}}_{i}\hat{x}\left(t\right)+{\bar{A}}_{di}\hat{x}\left(t-d\left(t\right)\right)\right]}^{T}R_{2}\left[{\bar{A}}_{i}\hat{x}\left(t\right)+{\bar{A}}_{di}\hat{x}\left(t-d\left(t\right)\right)\right] \end{array}$$

(46) $$\delta V\left(t\right)\leq \xi_{1}^{T}\left(t\right)\tilde{\Xi }\xi_{1}\left(t\right)$$

where

$$\xi_{1}^{T}\left(t\right)=\left[\begin{array}{ccccc} {\hat{x}}^{T}\left(t\right) & {\hat{x}}^{T}\left(t-d\left(t\right)\right)) & {\hat{x}}^{T}\left(t-d\right) & \frac{1}{d}\int_{t-d}^{t}{\hat{x}}^{T}\left(s\right)ds & \frac{1}{d^{2}}\int_{t-d}^{t}\int_{\theta }^{t}{\hat{x}}^{T}\left(s\right)dsd\theta \end{array}\right]$$

According to the Lyapunov principle, the sufficient conditions for the stochastic stability of UNSSUAFRS (25) under passive conditions are obtained as follows:

(47) $$\tilde{\Xi }=\left[\begin{array}{ccccc} \Omega_{11} & \Omega_{12} & 3{\bar{E}}^{T}R_{2}\bar{E} & -24{\bar{E}}^{T}R_{2}\bar{E} & 60{\bar{E}}^{T}R_{2}\bar{E}\\ {}* & \Omega_{22} & 0 & 0 & 0\\ {}* & * & \Omega_{33} & 36{\bar{E}}^{T}R_{2}\bar{E} & -60{\bar{E}}^{T}R_{2}\bar{E}\\ {}* & * & * & -4d^{2}R_{1}-192{\bar{E}}^{T}R_{2}\bar{E} & 6d^{2}R_{1}+360{\bar{E}}^{T}R_{2}\bar{E}\\ {}* & * & * & * & -12d^{2}R_{1}-720{\bar{E}}^{T}R_{2}\bar{E} \end{array}\right]<0$$

where

(48) $$\begin{array}{c} \Omega_{11}=sym\left\{{\left({\bar{A}}_{i}+\Delta {\bar{A}}_{i}\right)}^{T}\left(P_{i}\bar{E}+\bar{S}{\bar{R}}_{i}^{T}\right)\right\}+d^{2}{\left({\bar{A}}_{i}+\Delta {\bar{A}}_{i}\right)}^{T}R_{2}\left({\bar{A}}_{i}+\Delta {\bar{A}}_{i}\right)\\ \;+Q_{1}+Q_{2}+d^{2}R_{1}-9{\bar{E}}^{T}R_{2}\bar{E}+\sum_{j=1}^{N}\lambda_{ij}{\bar{E}}^{T}P_{j}\bar{E}\\ \Omega_{12}={\left({\bar{A}}_{di}+\Delta {\bar{A}}_{di}\right)}^{T}\left(P_{i}\bar{E}+\bar{S}{\bar{R}}_{i}^{T}\right)+d^{2}{\left({\bar{A}}_{di}+\Delta {\bar{A}}_{di}\right)}^{T}R_{2}\left({\bar{A}}_{di}+\Delta {\bar{A}}_{di}\right)\\ \Omega_{22}=-\left(1-\mu \right)Q_{2}\\ \Omega_{33}=-Q_{1}-9{\bar{E}}^{T}R_{2}\bar{E} \end{array}$$

When $\tilde{\Xi }<0$, it can be known by Lyapunov’s principle that the system (25) is randomly stable. According to Definition 1, it can be known that UNSSUAFRS (25) is randomly admissible under passive conditions. □

## 6. Random Admissibility Analysis of UNSSUAFRS

**Theorem** **3.**  *Given a scalar η>0, if there exists a symmetric positive definite matrix $P_{i}>0$, $Q_{1}>0$, $Q_{2}>0$, $R_{1}>0$, $R_{2}>0$ and an arbitrary column full-rank matrix S such that ${\bar{E}}^{T}\bar{S}=0$ holds the LMI applicable to any i,j∈I, then UNSSUAFRS (24) is randomly permissible under $H_{\infty }$ performance metrics γ.*

(49) $$\bar{\Xi }=\left[\begin{array}{cccccccccccc} \Omega_{1,1} & \Omega_{1,2} & \Omega_{1,3} & \Omega_{1,4} & \Omega_{1,5} & \Omega_{1,6} & d{\bar{A}}_{i} & \Omega_{1,8} & \Omega_{1,9} & 0 & 0 & {\bar{L}}_{i}^{T}\\ {}* & \Omega_{2,2} & 0 & 0 & 0 & 0 & d{\bar{A}}_{di}^{T} & \Omega_{2,8} & 0 & \Omega_{2,10} & 0 & {\bar{L}}_{di}^{T}\\ {}* & * & \Omega_{3,3} & 0 & 0 & 0 & d\Gamma_{1i}^{T} & 0 & 0 & 0 & \Omega_{3,11} & \Gamma_{2i}^{T}\\ {}* & * & * & \Omega_{4,4} & \Omega_{4,5} & \Omega_{4,6} & 0 & 0 & 0 & 0 & 0 & 0\\ {}* & * & * & * & \Omega_{5,5} & \Omega_{5,6} & 0 & 0 & 0 & 0 & 0 & 0\\ {}* & * & * & * & * & \Omega_{6,6} & 0 & 0 & 0 & 0 & 0 & 0\\ {}* & * & * & * & * & * & -R_{2}^{-1} & 0 & 0 & 0 & 0 & 0\\ {}* & * & * & * & * & * & * & -R_{2}^{-1} & 0 & 0 & 0 & 0\\ {}* & * & * & * & * & * & * & * & \Omega_{9,9} & 0 & 0 & 0\\ {}* & * & * & * & * & * & * & * & * & \Omega_{10,10} & 0 & 0\\ {}* & * & * & * & * & * & * & * & * & * & \Omega_{11,11} & 0\\ {}* & * & * & * & * & * & * & * & * & * & * & -I \end{array}\right]<0$$

*where*

(50) $$\begin{array}{c} \Omega_{1,1}=sym\left\{{\bar{A}}_{i}^{T}\left(P_{i}\bar{E}+\bar{S}{\bar{R}}_{i}^{T}\right)\right\}+Q_{1}+Q_{2}+d^{2}R_{1}-9{\bar{E}}^{T}R_{2}\bar{E}+\sum_{j=1}^{N}\lambda_{ij}{\bar{E}}^{T}P_{j}\bar{E}\\ \Omega_{1,2}={\bar{A}}_{di}^{T}\left(P_{i}\bar{E}+\bar{S}{\bar{R}}_{i}^{T}\right)\\ \Omega_{2,2}=-\left(1-\mu \right)Q_{2}\\ \Omega_{1,3}=\Gamma_{1i}^{T}\left(P_{i}\bar{E}+\bar{S}{\bar{R}}_{i}^{T}\right)\\ \Omega_{3,3}=-\gamma^{2}I\\ \Omega_{1,4}=3{\bar{E}}^{T}R_{2}\bar{E}\\ \Omega_{4,4}=-Q_{1}-9{\bar{E}}^{T}R_{2}\bar{E}\\ \Omega_{1,5}=-24{\bar{E}}^{T}R_{2}\bar{E}\\ \Omega_{4,5}=36{\bar{E}}^{T}R_{2}\bar{E}\\ \Omega_{5,5}=-4d^{2}R_{1}-192{\bar{E}}^{T}R_{2}\bar{E}\\ \Omega_{1,6}=60{\bar{E}}^{T}R_{2}\bar{E}\\ \Omega_{4,6}=-60{\bar{E}}^{T}R_{2}\bar{E}\\ \Omega_{5,6}=6d^{2}R_{1}+360{\bar{E}}^{T}R_{2}\bar{E}\\ \Omega_{6,6}=-12d^{2}R_{1}-720{\bar{E}}^{T}R_{2}\bar{E}\\ \Omega_{1,8}=d{\bar{N}}_{i}^{T}{\bar{M}}_{1i}^{T}B_{Ti}\\ \Omega_{2,8}=d{\bar{N}}_{i}^{T}{\bar{M}}_{2i}^{T}B_{Ti}\\ \Omega_{1,9}=\left[\begin{array}{ccccccc} {\bar{E}}^{T}P_{i}B_{Ti}{\bar{M}}_{1i} & {\bar{N}}_{i}^{T} & {\bar{A}}_{i}^{T}R_{2}B_{Ti}{\bar{M}}_{1i} & {\bar{N}}_{i}^{T} & {\bar{N}}_{i}^{T} & {\bar{A}}_{i}^{T}R_{2}B_{Ti}{\bar{M}}_{2i} & {\bar{N}}_{i}^{T} \end{array}\right]\\ \Omega_{9,9}=-diag\left(\begin{array}{ccccccc} \varepsilon_{1} & \varepsilon_{1}^{-1} & \varepsilon_{3} & \varepsilon_{3}^{-1} & \varepsilon_{5}^{-1} & \varepsilon_{6} & \varepsilon_{7}^{-1} \end{array}\right)\\ \Omega_{2,10}=\left[\begin{array}{ccccccc} {\bar{E}}^{T}P_{i}B_{Ti}{\bar{M}}_{2i} & {\bar{N}}_{i}^{T} & {\bar{A}}_{di}^{T}R_{2}B_{Ti}{\bar{M}}_{2i} & {\bar{N}}_{i}^{T} & {\bar{A}}_{di}^{T}R_{2}B_{Ti}{\bar{M}}_{1i} & {\bar{N}}_{i}^{T} & {\bar{N}}_{i}^{T} \end{array}\right]\\ \Omega_{10,10}=-diag\left(\begin{array}{ccccccc} \varepsilon_{2} & \varepsilon_{2}^{-1} & \varepsilon_{4} & \varepsilon_{4}^{-1} & \varepsilon_{5} & \varepsilon_{6}^{-1} & \varepsilon_{8}^{-1} \end{array}\right)\\ \Omega_{3,11}=\left[\begin{array}{cc} \Gamma_{1i}^{T}R_{2}B_{Ti}{\bar{M}}_{1i} & \Gamma_{1i}^{T}R_{2}B_{Ti}{\tilde{M}}_{2i} \end{array}\right]\\ \Omega_{11,11}=-diag\left(\begin{array}{cc} \varepsilon_{7} & \varepsilon_{8} \end{array}\right) \end{array}$$

**Proof.**  Firstly, it is necessary to prove the random admissibility of the active UNSSUAFRS system (24). As defined in Definition 1, the system (24) needs to satisfy random regularity and random pulse-free. Suppose there exists a pair of non-singular matrices $M_{i},N_{i}\in R^{n}$ such that:

(51) $$\left\{\begin{array}{c} M_{i}\bar{E}N_{i}=\left[\begin{array}{cc} I_{r} & 0_{r\times (n-r)}\\ 0_{(n-r)\times r} & 0_{\left(n-r)\times (n-r\right)} \end{array}\right]\\ M_{i}{\bar{A}}_{i}N_{i}=\left[\begin{array}{cc} A_{11i} & A_{12i}\\ A_{21i} & A_{22i} \end{array}\right]\\ M_{i}P_{i}N_{i}=\left[\begin{array}{cc} P_{11i} & P_{12i}\\ {}* & P_{22i} \end{array}\right] \end{array}\right.$$

From the establishment of $E^{T}P_{i}=EP_{i}^{T}\geq 0$, we can obtain $A_{22i}^{T}P_{22i}+A_{22i}P_{22i}^{T}<0$. Therefore, it can be known that $A_{22i}$ is reversible, so the system is randomly regular and randomly pulse-free. To prove the stochastic stability of the system, the corresponding modal-dependent Lyapunov function was established:

(52) $$V\left(t\right)=V_{1}\left(t\right)+V_{2}\left(t\right)+V_{3}\left(t\right)+V_{4}\left(t\right)$$

where

(53) V1(t)=xT(t)E¯TPiE¯x(t)V2(t)=∫t−dtx^T(s)Q1x^(s)ds+∫t−d(t)tx^T(s)Q2x^(s)dsV3(t)=d∫−d0∫t+θtx^T(s)R1x^(s)dsdθV4(t)=d∫−d0∫t+θtx^˙T(s)E¯TR2E¯x^˙(s)dsdθ

By performing a weak infinitesimal operation on (53), we obtain:

(54) δV1(t)=x^˙T(t)E¯TPiE¯x^˙(t)+x^T(t)E¯TPiE¯x^˙(t)+x^T(t)∑j=1NλijE¯TPjE¯x^(t)=(A¯i+ΔA¯i)x^(t)+(A¯di+ΔA¯di)x^(t−d(t))TPiE¯x^(t)+x^T(t)E¯TPi(A¯i+ΔA¯)x^(t)+(A¯di+ΔA¯di)x^(t−d(t))+x^T(t)∑j=1NλijE¯TPjE¯x^(t)

(55) $$\begin{array}{c} \delta V_{2}\left(t\right)\leq {\hat{x}}^{T}\left(t\right)\left(Q_{1}+Q_{2}\right)\hat{x}\left(t\right)-{\hat{x}}^{T}\left(t-d\right)Q_{1}\hat{x}\left(t-d\right)\\ -\left(1-u\right){\hat{x}}^{T}\left(t-d\left(t\right)\right)Q_{2}\hat{x}\left(t-d\left(t\right)\right) \end{array}$$

(56) $$\delta V_{3}\left(t\right)=d^{2}{\hat{x}}^{T}\left(t\right)R_{1}\hat{x}\left(t\right)-d\int_{t-d}^{t}{\hat{x}}^{T}\left(s\right)R_{1}\hat{x}\left(s\right)ds$$

(57) δV4(t)=d2x^˙T(t)E¯TR2E¯x^˙(t)−d∫t−dtx^˙(s)E¯TR2E¯x^˙T(s)ds=d2(A¯i+ΔA¯i)x^(t)+(A¯di+ΔA¯di)x^(t−d(t))+Γ1iφ(t)TR2(A¯i+ΔA¯i)x^(t)+(A¯di+ΔA¯di)x^(t−d(t))+Γ1iφ(t)−d∫t−dtx^˙(s)E¯TR2E¯x^˙T(s)ds

When ${\bar{E}}^{T}\bar{S}=0$, the following zero equation applicable to any matrix can be derived:

(58) Λx^(t)=x^˙(t)E¯TS¯R¯iTx^(t)+x^(t)R¯iS¯TE¯x^˙(t)=A¯ix^(t)+A¯dix^(t−d(t))+Γ1iφ(t)TS¯R¯iTx^(t)+x^(t)R¯iS¯TA¯ix^(t)+A¯dix^(t−d(t))+Γ1iφ(t)=0

Add the Formulas (54)–(58) to obtain the weak infinitesimal operator δV(t):

(59) $$\begin{array}{c} \delta V\left(t\right)\leq {\left[\left({\bar{A}}_{i}+\Delta {\bar{A}}_{i}\right)\hat{x}\left(t\right)+\left({\bar{A}}_{di}+\Delta {\bar{A}}_{di}\right)\hat{x}\left(t-d\left(t\right)\right)+\Gamma_{1i}\varphi \left(t\right)\right]}^{T}P_{i}\bar{E}\hat{x}\left(t\right)\\ +{\hat{x}}^{T}\left(t\right){\bar{E}}^{T}P_{i}\left[\left({\bar{A}}_{i}+\Delta {\bar{A}}_{i}\right)\hat{x}\left(t\right)+{\bar{A}}_{di}+\Delta {\bar{A}}_{di}\hat{x}\left(t-d\left(t\right)\right)+\Gamma_{1i}\varphi \left(t\right)\right]\\ +{\hat{x}}^{T}\left(t\right)\sum_{j=1}^{N}\lambda_{ij}{\bar{E}}^{T}P_{j}\bar{E}\hat{x}\left(t\right)+{\left[{\bar{A}}_{i}\hat{x}\left(t\right)+{\bar{A}}_{di}\hat{x}\left(t-d\left(t\right)\right)+\Gamma_{1i}\varphi \left(t\right)\right]}^{T}\bar{S}{\bar{R}}_{i}^{T}\hat{x}\left(t\right)\\ +\hat{x}\left(t\right){\bar{R}}_{i}{\bar{S}}^{T}\left[{\bar{A}}_{i}\hat{x}\left(t\right)+{\bar{A}}_{di}\hat{x}\left(t-d\left(t\right)\right)+\Gamma_{1i}\varphi \left(t\right)\right]+{\hat{x}}^{T}\left(t\right)\left(Q_{1}+Q_{2}\right)\hat{x}\left(t\right)\\ -{\hat{x}}^{T}\left(t-d\right)Q_{1}\hat{x}\left(t-d\right)-\left(1-\mu \right){\hat{x}}^{T}\left(t-d\left(t\right)\right)Q_{2}\hat{x}\left(t-d\left(t\right)\right)\\ -9{\hat{x}}^{T}\left(t\right){\bar{E}}^{T}R_{2}\bar{E}\hat{x}\left(t\right)-24{\hat{x}}^{T}\left(t\right){\bar{E}}^{T}R_{2}\bar{E}\int_{t-d}^{t}\hat{x}\left(s\right)ds\\ +{\hat{x}}^{T}\left(t-d\right)\left(36{\bar{E}}^{T}R_{2}\bar{E}\right)\int_{t-d}^{t}\hat{x}\left(s\right)ds+{\hat{x}}^{T}\left(t\right)\left(60{\bar{E}}^{T}R_{2}\bar{E}\right)\int_{t-d}^{t}\int_{\theta }^{t}\hat{x}\left(s\right)dsd\theta \\ +{\hat{x}}^{T}\left(t\right)\left(-60{\bar{E}}^{T}R_{2}\bar{E}\right)\int_{t-d}^{t}\int_{\theta }^{t}\hat{x}\left(s\right)dsd\theta +d{\hat{x}}^{T}\left(t\right)R_{1}\hat{x}\left(t\right)\\ -\int_{t-d}^{t}{\hat{x}}^{T}\left(s\right)ds\left(-4d^{2}R_{1}-192{\bar{E}}^{T}R_{2}\bar{E}\right)\int_{t-d}^{t}\hat{x}\left(s\right)ds\\ +\int_{t-d}^{t}{\hat{x}}^{T}\left(s\right)ds\left(6d^{2}R_{1}+360{\bar{E}}^{T}R_{2}\bar{E}\right)\int_{t-d}^{t}\int_{\theta }^{t}{\hat{x}}^{T}\left(s\right)dsd\theta \\ +\int_{t-d}^{t}\int_{\theta }^{t}{\hat{x}}^{T}\left(s\right)dsd\theta \left(-12d^{2}R_{1}-720{\bar{E}}^{T}R_{2}\bar{E}\right)\int_{t-d}^{t}\int_{\theta }^{t}\hat{x}\left(s\right)dsd\theta \\ +d^{2}{\left[\left({\bar{A}}_{i}+\Delta {\bar{A}}_{i}\right)\hat{x}\left(t\right)+\left({\bar{A}}_{di}+\Delta {\bar{A}}_{di}\right)\hat{x}\left(t-d\left(t\right)\right)+\Gamma_{1i}\varphi \left(t\right)\right]}^{T}\\ R_{2}\left[\left({\bar{A}}_{i}+\Delta {\bar{A}}_{i}\right)\hat{x}\left(t\right)+\left({\bar{A}}_{di}+\Delta {\bar{A}}_{di}\right)\hat{x}\left(t-d\left(t\right)\right)+\Gamma_{1i}\varphi \left(t\right)\right] \end{array}$$

According to Lemmas 3 and 4, the uncertain parameters $\Delta {\bar{A}}_{i}$ and $Delta{\bar{A}}_{di}$ existing in Equation (59) are determined. Therefore, the piecewise formula in Formula (59) is transformed into the following form:

(60) $$\begin{array}{c} {\hat{x}}^{T}\left(t\right)\Delta {\bar{A}}_{i}^{T}B_{Ti}^{T}P_{i}\bar{E}\hat{x}\left(t\right)+{\hat{x}}^{T}\left(t\right){\bar{E}}^{T}P_{i}B_{Ti}\Delta {\tilde{A}}_{i}\hat{x}\left(t\right)\\ =\varepsilon_{1}{\hat{x}}^{T}\left(t\right){\bar{E}}^{T}P_{i}B_{Ti}{\bar{M}}_{1i}{\bar{M}}_{1i}^{T}B_{Ti}^{T}P_{i}\bar{E}\hat{x}\left(t\right)+\varepsilon_{1}^{-1}{\hat{x}}^{T}\left(t\right){\bar{N}}_{i}^{T}{\bar{N}}_{i}\hat{x}\left(t\right) \end{array}$$

(61) $$\begin{array}{c} {\hat{x}}^{T}\left(t-d\left(t\right)\right)\Delta {\bar{A}}_{di}^{T}B_{Ti}^{T}P_{i}\bar{E}\hat{x}\left(t-d\left(t\right)\right)+{\hat{x}}^{T}\left(t-d\left(t\right)\right){\bar{E}}^{T}P_{i}B_{Ti}\Delta {\bar{A}}_{di}\hat{x}\left(t-d\left(t\right)\right)\\ =\varepsilon_{2}{\hat{x}}^{T}\left(t-d\left(t\right)\right){\tilde{E}}^{T}P_{i}B_{Ti}{\bar{M}}_{2i}{\bar{M}}_{2i}^{T}B_{Ti}^{T}P_{i}\tilde{E}\hat{x}\left(t-d\left(t\right)\right)+\varepsilon_{2}^{-1}{\hat{x}}^{T}\left(t-d\left(t\right)\right){\bar{N}}_{i}^{T}{\bar{N}}_{i}\hat{x}\left(t-d\left(t\right)\right) \end{array}$$

(62) $$\begin{array}{c} {\hat{x}}^{T}\left(t\right)\Delta {\bar{A}}_{i}^{T}B_{Ti}^{T}R_{2}{\bar{A}}_{i}\hat{x}\left(t\right)+{\hat{x}}^{T}\left(t\right){\bar{A}}_{i}^{T}{R_{2}}_{i}B_{Ti}\Delta {\bar{A}}_{i}\hat{x}\left(t\right)\\ =\varepsilon_{3}{\hat{x}}^{T}\left(t\right){\bar{A}}_{i}^{T}R_{2}B_{Ti}{\bar{M}}_{1i}{\bar{M}}_{1i}^{T}B_{Ti}^{T}R_{2}{\bar{A}}_{i}\hat{x}\left(t\right)+\varepsilon_{3}^{-1}{\hat{x}}^{T}\left(t\right){\bar{N}}_{i}^{T}{\bar{N}}_{i}\hat{x}\left(t\right) \end{array}$$

(63) $$\begin{array}{c} {\hat{x}}^{T}\left(t-d\left(t\right)\right){\bar{A}}_{di}^{T}R_{2}B_{Ti}\Delta {\bar{A}}_{di}\hat{x}\left(t-d\left(t\right)\right)+{\hat{x}}^{T}\left(t-d\left(t\right)\right)\Delta {\bar{A}}_{di}^{T}B_{Ti}^{T}R_{2}{\bar{A}}_{di}\hat{x}\left(t\right)\\ =\varepsilon_{4}{\hat{x}}^{T}\left(t-d\left(t\right)\right){\bar{A}}_{di}^{T}R_{2}B_{Ti}{\tilde{M}}_{2i}{\tilde{M}}_{2i}^{T}B_{Ti}^{T}R_{2}{\bar{A}}_{di}\hat{x}\left(t-d\left(t\right)\right)\\ +\varepsilon_{4}^{-1}{\hat{x}}^{T}\left(t-d\left(t\right)\right){\bar{N}}_{i}^{T}{\bar{N}}_{i}\hat{x}\left(t-d\left(t\right)\right) \end{array}$$

(64) $$\begin{array}{c} {\hat{x}}^{T}\left(t\right)\Delta {\bar{A}}_{i}^{T}B_{Ti}^{T}R_{2}{\bar{A}}_{di}\hat{x}\left(t-d\left(t\right)\right)+{\hat{x}}^{T}\left(t-d\left(t\right)\right){\bar{A}}_{di}^{T}{R_{2}}_{i}B_{Ti}\Delta {\bar{A}}_{i}\hat{x}\left(t\right)\\ =\varepsilon_{5}{\hat{x}}^{T}\left(t-d\left(t\right)\right){\bar{A}}_{di}^{T}R_{2}B_{Ti}{\bar{M}}_{1i}{\bar{M}}_{1i}^{T}B_{Ti}^{T}R_{2}{\bar{A}}_{di}\hat{x}\left(t-d\left(t\right)\right)+\varepsilon_{5}^{-1}{\hat{x}}^{T}\left(t\right){\bar{N}}_{i}^{T}{\bar{N}}_{i}\hat{x}\left(t\right) \end{array}$$

(65) $$\begin{array}{c} {\hat{x}}^{T}\left(t\right){\bar{A}}_{i}^{T}R_{2}B_{Ti}\Delta {\bar{A}}_{di}\hat{x}\left(t-d\left(t\right)\right)+{\hat{x}}^{T}\left(t-d\left(t\right)\right)\Delta {\bar{A}}_{di}^{T}B_{Ti}^{T}R_{2}{\bar{A}}_{i}\hat{x}\left(t\right)\\ =\varepsilon_{6}{\hat{x}}^{T}\left(t\right){\bar{A}}_{i}^{T}R_{2}B_{Ti}{\tilde{M}}_{2i}{\tilde{M}}_{2i}^{T}B_{Ti}^{T}R_{2}{\bar{A}}_{i}\hat{x}\left(t\right)+\varepsilon_{6}^{-1}{\hat{x}}^{T}\left(t-d\left(t\right)\right){\bar{N}}_{i}^{T}{\bar{N}}_{i}\hat{x}\left(t-d\left(t\right)\right) \end{array}$$

(66) $$\begin{array}{c} {\hat{x}}^{T}\left(t\right)\Delta {\bar{A}}_{i}^{T}B_{Ti}^{T}R_{2}\Gamma_{1i}\varphi \left(t\right)+\varphi^{T}\left(t\right)\Gamma_{1i}^{T}{R_{2}}_{i}B_{Ti}\Delta {\bar{A}}_{i}\hat{x}\left(t\right)\\ =\varepsilon_{7}\varphi^{T}\left(t\right)\Gamma_{1i}^{T}R_{2}B_{Ti}{\bar{M}}_{1i}{\bar{M}}_{1i}^{T}B_{Ti}^{T}R_{2}\Gamma_{1i}\varphi \left(t\right)+\varepsilon_{7}^{-1}{\hat{x}}^{T}\left(t\right){\bar{N}}_{i}^{T}{\bar{N}}_{i}\hat{x}\left(t\right) \end{array}$$

(67) $$\begin{array}{c} \varphi^{T}\left(t\right)\Gamma_{1i}^{T}R_{2}B_{Ti}\Delta {\bar{A}}_{di}\hat{x}\left(t-d\left(t\right)\right)+{\hat{x}}^{T}\left(t-d\left(t\right)\right)\Delta {\bar{A}}_{di}^{T}B_{Ti}^{T}R_{2}\Gamma_{1i}\varphi \left(t\right)\\ =\varepsilon_{8}\varphi^{T}\left(t\right)\Gamma_{1i}^{T}R_{2}B_{Ti}{\tilde{M}}_{2i}{\tilde{M}}_{2i}^{T}B_{Ti}^{T}R_{2}\Gamma_{1i}\varphi \left(t\right)+\varepsilon_{8}^{-1}{\hat{x}}^{T}\left(t-d\left(t\right)\right){\bar{N}}_{i}^{T}{\bar{N}}_{i}\hat{x}\left(t-d\left(t\right)\right) \end{array}$$

According to Definition 2, for t>0, there exists a scalar γ>0 that can ensure the $H_{\infty }$ performance of the fault detection filter under this metric, and the following formula is obtained:

(68) $$\begin{array}{c} J\left(T\right)=E\left\{\int_{0}^{T}\left[r^{T}\left(t\right)r\left(t\right)-\gamma^{2}\varphi^{T}\left(t\right)\varphi \left(t\right)\right]dt\right\}\\ =E\left\{\int_{0}^{T}\left[r^{T}\left(t\right)r\left(t\right)-\gamma^{2}\varphi^{T}\left(t\right)\varphi \left(t\right)+\delta V\left(x\left(t\right)\right)\right]ds\right\}-E\left[\delta V(x(t))\right]\\ \leq E\left\{\int_{0}^{T}\left[r^{T}\left(t\right)r\left(t\right)-\gamma^{2}\varphi^{T}\left(t\right)\varphi \left(t\right)+\delta V\left(x\left(t\right)\right)\right]ds\right\} \end{array}$$

Substituting r(t) Formula (24) into Formula (68) and combining it with Formulas (59)–(67), we can obtain:

(69) $$\begin{array}{c} J\left(T\right)\leq {\left[{\bar{A}}_{i}\hat{x}\left(t\right)+{\bar{A}}_{di}\hat{x}\left(t-d\left(t\right)\right)+\Gamma_{1i}\varphi \left(t\right)\right]}^{T}P_{i}\bar{E}\hat{x}\left(t\right)\\ +{\hat{x}}^{T}\left(t\right){\bar{E}}^{T}P_{i}\left[{\bar{A}}_{i}\hat{x}\left(t\right)+{\bar{A}}_{di}\hat{x}\left(t-d\left(t\right)\right)+\Gamma_{1i}\varphi \left(t\right)\right]\\ +{\hat{x}}^{T}\left(t\right)\sum_{j=1}^{N}\lambda_{ij}{\bar{E}}^{T}P_{j}\bar{E}\hat{x}\left(t\right)+{\hat{x}}^{T}\left(t\right)\left(Q_{1}+Q_{2}\right)\hat{x}\left(t\right)\\ -{\hat{x}}^{T}\left(t-d\right)Q_{1}\hat{x}\left(t-d\right)-\left(1-\mu \right){\hat{x}}^{T}\left(t-d\left(t\right)\right)Q_{2}\hat{x}\left(t-d\left(t\right)\right)\\ -9{\hat{x}}^{T}\left(t\right){\bar{E}}^{T}R_{2}\bar{E}\hat{x}\left(t\right)-24{\hat{x}}^{T}\left(t\right){\bar{E}}^{T}R_{2}\bar{E}\int_{t-d}^{t}\hat{x}\left(s\right)ds\\ +{\hat{x}}^{T}\left(t-d\right)\left(36{\bar{E}}^{T}R_{2}\bar{E}\right)\int_{t-d}^{t}\hat{x}\left(s\right)ds+{\hat{x}}^{T}\left(t\right)\left(60{\bar{E}}^{T}R_{2}\bar{E}\right)\int_{t-d}^{t}\int_{\theta }^{t}\hat{x}\left(s\right)dsd\theta \\ +{\hat{x}}^{T}\left(t\right)\left(-60{\bar{E}}^{T}R_{2}\bar{E}\right)\int_{t-d}^{t}\int_{\theta }^{t}\hat{x}\left(s\right)dsd\theta +d{\hat{x}}^{T}\left(t\right)R_{1}\hat{x}\left(t\right)\\ -\int_{t-d}^{t}{\hat{x}}^{T}\left(s\right)ds\left(-4d^{2}R_{1}-192{\bar{E}}^{T}R_{2}\bar{E}\right)\int_{t-d}^{t}\hat{x}\left(s\right)ds\\ +\int_{t-d}^{t}{\hat{x}}^{T}\left(s\right)ds\left(6d^{2}R_{1}+360{\bar{E}}^{T}R_{2}\bar{E}\right)\int_{t-d}^{t}\int_{\theta }^{t}{\hat{x}}^{T}\left(s\right)dsd\theta \\ +\int_{t-d}^{t}\int_{\theta }^{t}{\hat{x}}^{T}\left(s\right)dsd\theta \left(-12d^{2}R_{1}-720{\bar{E}}^{T}R_{2}\bar{E}\right)\int_{t-d}^{t}\int_{\theta }^{t}\hat{x}\left(s\right)dsd\theta \\ +d^{2}{\left[{\bar{A}}_{i}\hat{x}\left(t\right)+{\bar{A}}_{di}\hat{x}\left(t-d\left(t\right)\right)+\Gamma_{1i}\varphi \left(t\right)\right]}^{T}R_{2}\left[{\bar{A}}_{i}\hat{x}\left(t\right)+{\bar{A}}_{di}\hat{x}\left(t-d\left(t\right)\right)+\Gamma_{1i}\varphi \left(t\right)\right]\\ +{\left[{\bar{L}}_{i}\hat{x}\left(t\right)+{\bar{L}}_{di}\hat{x}\left(t-d\left(t\right)\right)+\Gamma_{2i}\varphi \left(t\right)\right]}^{T}\left[{\bar{L}}_{i}\hat{x}\left(t\right)+{\bar{L}}_{di}\hat{x}\left(t-d\left(t\right)\right)+\Gamma_{2i}\varphi \left(t\right)\right]\\ +\varepsilon_{1}{\hat{x}}^{T}\left(t\right){\bar{E}}^{T}P_{i}B_{Ti}{\bar{M}}_{1i}{\bar{M}}_{1i}^{T}B_{Ti}^{T}P_{i}\bar{E}\hat{x}\left(t\right)+\varepsilon_{1}^{-1}{\hat{x}}^{T}\left(t\right){\bar{N}}_{i}^{T}{\bar{N}}_{i}\hat{x}\left(t\right)\\ +\varepsilon_{2}{\hat{x}}^{T}\left(t-d\left(t\right)\right){\tilde{E}}^{T}P_{i}B_{Ti}{\bar{M}}_{2i}{\bar{M}}_{2i}^{T}B_{Ti}^{T}P_{i}\tilde{E}\hat{x}\left(t-d\left(t\right)\right)\\ +\varepsilon_{2}^{-1}{\hat{x}}^{T}\left(t-d\left(t\right)\right){\bar{N}}_{i}^{T}{\bar{N}}_{i}\hat{x}\left(t-d\left(t\right)\right)+\varepsilon_{3}{\hat{x}}^{T}\left(t\right){\bar{A}}_{i}^{T}R_{2}B_{Ti}{\bar{M}}_{1i}{\bar{M}}_{1i}^{T}B_{Ti}^{T}R_{2}{\bar{A}}_{i}\hat{x}\left(t\right)\\ +\varepsilon_{3}^{-1}{\hat{x}}^{T}\left(t\right){\bar{N}}_{i}^{T}{\bar{N}}_{i}\hat{x}\left(t\right)+\varepsilon_{4}{\hat{x}}^{T}\left(t-d\left(t\right)\right){\bar{A}}_{di}^{T}R_{2}B_{Ti}{\tilde{M}}_{2i}{\tilde{M}}_{2i}^{T}B_{Ti}^{T}R_{2}{\bar{A}}_{di}\hat{x}\left(t-d\left(t\right)\right)\\ +\varepsilon_{4}^{-1}{\hat{x}}^{T}\left(t-d\left(t\right)\right){\bar{N}}_{i}^{T}{\bar{N}}_{i}\hat{x}\left(t-d\left(t\right)\right)+\varepsilon_{5}{\hat{x}}^{T}\left(t-d\left(t\right)\right){\bar{A}}_{di}^{T}R_{2}B_{Ti}{\bar{M}}_{1i}{\bar{M}}_{1i}^{T}B_{Ti}^{T}R_{2}{\bar{A}}_{di}\hat{x}\left(t-d\left(t\right)\right)\\ +\varepsilon_{5}^{-1}{\hat{x}}^{T}\left(t\right){\bar{N}}_{i}^{T}{\bar{N}}_{i}\hat{x}\left(t\right)+\varepsilon_{6}{\hat{x}}^{T}\left(t\right){\bar{A}}_{i}^{T}R_{2}B_{Ti}{\tilde{M}}_{2i}{\tilde{M}}_{2i}^{T}B_{Ti}^{T}R_{2}{\bar{A}}_{i}\hat{x}\left(t\right)\\ +\varepsilon_{6}^{-1}{\hat{x}}^{T}\left(t-d\left(t\right)\right){\bar{N}}_{i}^{T}{\bar{N}}_{i}\hat{x}\left(t-d\left(t\right)\right)+\varepsilon_{7}\varphi^{T}\left(t\right)\Gamma_{1i}^{T}R_{2}B_{Ti}{\bar{M}}_{1i}{\bar{M}}_{1i}^{T}B_{Ti}^{T}R_{2}\Gamma_{1i}\varphi \left(t\right)\\ +\varepsilon_{7}^{-1}{\hat{x}}^{T}\left(t\right){\bar{N}}_{i}^{T}{\bar{N}}_{i}\hat{x}\left(t\right)+\varepsilon_{8}\varphi^{T}\left(t\right)\Gamma_{1i}^{T}R_{2}B_{Ti}{\tilde{M}}_{2i}{\tilde{M}}_{2i}^{T}B_{Ti}^{T}R_{2}\Gamma_{1i}\varphi \left(t\right)\\ +\varepsilon_{8}^{-1}{\hat{x}}^{T}\left(t-d\left(t\right)\right){\bar{N}}_{i}^{T}{\bar{N}}_{i}\hat{x}\left(t-d\left(t\right)\right)-\gamma^{2}\varphi^{T}\left(t\right)\varphi \left(t\right) \end{array}$$

(70) $$J\left(T\right)\leq E\left\{\int_{0}^{T}\left[{\xi_{2}}^{T}\left(t\right)\bar{\Xi }\xi_{2}\left(t\right)\right]dt\right\}$$

where

$${\xi_{2}}^{T}\left(t\right)=\left[\begin{array}{cccccc} {\hat{x}}^{T}\left(t\right) & {\hat{x}}^{T}\left(t-d\left(t\right)\right) & \varphi^{T}\left(t\right) & {\hat{x}}^{T}\left(t-d\right) & \frac{1}{d}\int_{t-d}^{t}{\hat{x}}^{T}\left(s\right)ds & \frac{1}{d^{2}}\int_{t-d}^{t}\int_{\theta }^{t}{\hat{x}}^{T}\left(s\right)dsd\theta \end{array}\right]$$

By performing the Schur complement operation on $\bar{\Xi }$, we can obtain:

(71) $$\bar{\Xi }=\left[\begin{array}{cccccccccccc} \Omega_{1,1} & \Omega_{1,2} & \Omega_{1,3} & \Omega_{1,4} & \Omega_{1,5} & \Omega_{1,6} & d{\bar{A}}_{i} & \Omega_{1,8} & \Omega_{1,9} & 0 & 0 & {\bar{L}}_{i}^{T}\\ {}* & \Omega_{2,2} & 0 & 0 & 0 & 0 & d{\bar{A}}_{di}^{T} & \Omega_{2,8} & 0 & \Omega_{2,10} & 0 & {\bar{L}}_{di}^{T}\\ {}* & * & \Omega_{3,3} & 0 & 0 & 0 & d\Gamma_{1i}^{T} & 0 & 0 & 0 & \Omega_{3,11} & \Gamma_{2i}^{T}\\ {}* & * & * & \Omega_{4,4} & \Omega_{4,5} & \Omega_{4,6} & 0 & 0 & 0 & 0 & 0 & 0\\ {}* & * & * & * & \Omega_{5,5} & \Omega_{5,6} & 0 & 0 & 0 & 0 & 0 & 0\\ {}* & * & * & * & * & \Omega_{6,6} & 0 & 0 & 0 & 0 & 0 & 0\\ {}* & * & * & * & * & * & -R_{2}^{-1} & 0 & 0 & 0 & 0 & 0\\ {}* & * & * & * & * & * & * & -R_{2}^{-1} & 0 & 0 & 0 & 0\\ {}* & * & * & * & * & * & * & * & \Omega_{9,9} & 0 & 0 & 0\\ {}* & * & * & * & * & * & * & * & * & \Omega_{10,10} & 0 & 0\\ {}* & * & * & * & * & * & * & * & * & * & \Omega_{11,11} & 0\\ {}* & * & * & * & * & * & * & * & * & * & * & -I \end{array}\right]$$

where

$$\begin{array}{c} \Omega_{1,1}=sym\left\{{\bar{A}}_{i}^{T}\left(P_{i}\bar{E}+\bar{S}{\bar{R}}_{i}^{T}\right)\right\}+Q_{1}+Q_{2}+d^{2}R_{1}-9{\bar{E}}^{T}R_{2}\bar{E}+\sum_{j=1}^{N}\lambda_{ij}{\bar{E}}^{T}P_{j}\bar{E}\\ \Omega_{1,2}={\bar{A}}_{di}^{T}\left(P_{i}\bar{E}+\bar{S}{\bar{R}}_{i}^{T}\right)\\ \Omega_{2,2}=-\left(1-\mu \right)Q_{2}\\ \Omega_{1,3}=\Gamma_{1i}^{T}\left(P_{i}\bar{E}+\bar{S}{\bar{R}}_{i}^{T}\right)\\ \Omega_{3,3}=-\gamma^{2}I\\ \Omega_{1,4}=3{\bar{E}}^{T}R_{2}\bar{E}\\ \Omega_{4,4}=-Q_{1}-9{\bar{E}}^{T}R_{2}\bar{E}\\ \Omega_{1,5}=-24{\bar{E}}^{T}R_{2}\bar{E}\\ \Omega_{4,5}=36{\bar{E}}^{T}R_{2}\bar{E}\\ \Omega_{5,5}=-4d^{2}R_{1}-192{\bar{E}}^{T}R_{2}\bar{E}\\ \Omega_{1,6}=60{\bar{E}}^{T}R_{2}\bar{E}\\ \Omega_{4,6}=-60{\bar{E}}^{T}R_{2}\bar{E}\\ \Omega_{5,6}=6d^{2}R_{1}+360{\bar{E}}^{T}R_{2}\bar{E}\\ \Omega_{6,6}=-12d^{2}R_{1}-720{\bar{E}}^{T}R_{2}\bar{E}\\ \Omega_{1,8}=d{\bar{N}}_{i}^{T}{\bar{M}}_{1i}^{T}B_{Ti}\\ \Omega_{2,8}=d{\bar{N}}_{i}^{T}{\bar{M}}_{2i}^{T}B_{Ti}\\ \Omega_{1,9}=\left[\begin{array}{ccccccc} {\bar{E}}^{T}P_{i}B_{Ti}{\bar{M}}_{1i} & {\bar{N}}_{i}^{T} & {\bar{A}}_{i}^{T}R_{2}B_{Ti}{\bar{M}}_{1i} & {\bar{N}}_{i}^{T} & {\bar{N}}_{i}^{T} & {\bar{A}}_{i}^{T}R_{2}B_{Ti}{\bar{M}}_{2i} & {\bar{N}}_{i}^{T} \end{array}\right]\\ \Omega_{9,9}=-diag\left(\begin{array}{ccccccc} \varepsilon_{1} & \varepsilon_{1}^{-1} & \varepsilon_{3} & \varepsilon_{3}^{-1} & \varepsilon_{5}^{-1} & \varepsilon_{6} & \varepsilon_{7}^{-1} \end{array}\right)\\ \Omega_{2,10}=\left[\begin{array}{ccccccc} {\bar{E}}^{T}P_{i}B_{Ti}{\bar{M}}_{2i} & {\bar{N}}_{i}^{T} & {\bar{A}}_{di}^{T}R_{2}B_{Ti}{\bar{M}}_{2i} & {\bar{N}}_{i}^{T} & {\bar{A}}_{di}^{T}R_{2}B_{Ti}{\bar{M}}_{1i} & {\bar{N}}_{i}^{T} & {\bar{N}}_{i}^{T} \end{array}\right]\\ \Omega_{10,10}=-diag\left(\begin{array}{ccccccc} \varepsilon_{2} & \varepsilon_{2}^{-1} & \varepsilon_{4} & \varepsilon_{4}^{-1} & \varepsilon_{5} & \varepsilon_{6}^{-1} & \varepsilon_{8}^{-1} \end{array}\right)\\ \Omega_{3,11}=\left[\begin{array}{cc} \Gamma_{1i}^{T}R_{2}B_{Ti}{\bar{M}}_{1i} & \Gamma_{1i}^{T}R_{2}B_{Ti}{\tilde{M}}_{2i} \end{array}\right]\\ \Omega_{11,11}=-diag\left(\begin{array}{cc} \varepsilon_{7} & \varepsilon_{8} \end{array}\right) \end{array}$$

When $\bar{\Xi }<0$, according to the Lyapunov principle, the system is stable. Combined with Definition 1, it can be proved that UNSSUAFRS (24) is randomly permissible under active conditions. Thus, the proof of Theorem 3 is completed.

**Remark** **4.**  *System (24) is randomly permissible when Theorem 3 is satisfied. Under this condition, its strict LMIs can be solved through Matlab, and the parameters of the designed fault detection filter can be obtained.* □

## 7. Simulation Analysis

In this section, the practicality and validity of the obtained results are verified by using the DC servo motor model, numerical examples and the F404 aircraft model.

**Figure 1:**
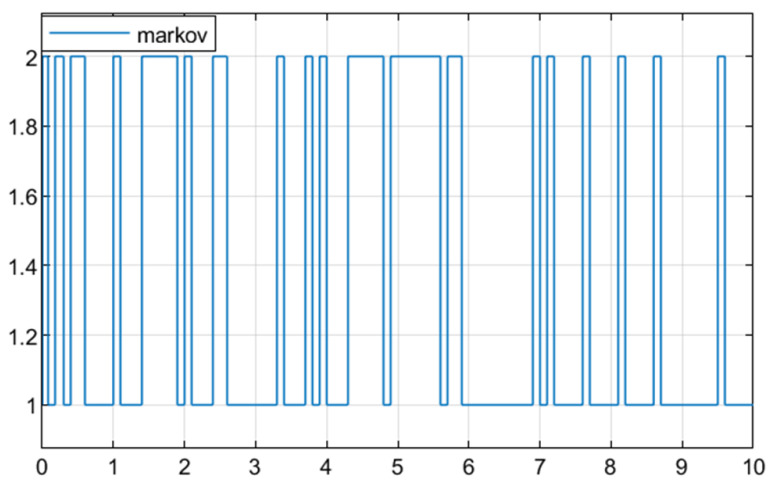
The Markov process.

**Example** **1.**  *In this section, numerical simulation analysis is conducted on system (2) based on Theorem 3 to verify the effectiveness of the designed fault detection filter (23). Let *   $E=\left[\begin{array}{cc} 1 & 0\\ 0 & 0 \end{array}\right]$*,*  $S={\left[\begin{array}{cc} 0 & 1 \end{array}\right]}^{T}$*,*  $\lambda_{ij}=\left[\begin{array}{cc} -0.8 & 0.8\\ 0.6 & -0.6 \end{array}\right]$*, where Figure 1 shows the Markov process at this transfer rate. The parameters of the system are described as follows:*

*When*  i=1:

$A\left\{1\right\}=\left[\begin{array}{cc} 2 & -1\\ 1 & 2 \end{array}\right]$, $A_{d}\left\{1\right\}=\left[\begin{array}{cc} 0 & 0\\ 0.5 & 0 \end{array}\right]$, $B\left\{1\right\}=\left[\begin{array}{c} 1\\ 1 \end{array}\right]$, $C\left\{1\right\}=\left[1.\begin{array}{cc} 2 & 0.6 \end{array}\right]$, $C_{d}\left\{1\right\}=\left[\begin{array}{cc} 0.15 & 0.15 \end{array}\right]$, $D\left\{1\right\}=0.3$, $F\left\{1\right\}=\left[\begin{array}{c} 0.6\\ 0.9 \end{array}\right]$, $G\left\{1\right\}=0.1$, $H\left\{1\right\}=\left[\begin{array}{c} 0.5\\ 0.2 \end{array}\right]$, $L\left\{1\right\}=\left[\begin{array}{cc} 2 & 1 \end{array}\right]$, $L_{d}\left\{1\right\}=\left[\begin{array}{cc} 0.2 & 0.1 \end{array}\right]$, $T\left\{1\right\}=\left[\begin{array}{cc} 1 & 1 \end{array}\right]$, $M_{1}\left\{1\right\}=\left[\begin{array}{cc} 0.5 & 0\\ 0 & 0.1 \end{array}\right]$, $M_{2}\left\{1\right\}=\left[\begin{array}{cc} 0.5 & 0\\ 0 & 0.1 \end{array}\right]$, $N\left\{1\right\}=\left[\begin{array}{cc} 0.3 & 0\\ 0 & 0.3 \end{array}\right]$.

*When*  i=2:

$A\left\{2\right\}=\left[\begin{array}{cc} 1 & 2\\ -1 & 0.5 \end{array}\right]$, $A_{d}\left\{2\right\}=\left[\begin{array}{cc} 0 & 0\\ 0.5 & 0 \end{array}\right]$, $B\left\{2\right\}=\left[\begin{array}{c} 1.3\\ 1 \end{array}\right]$, $C\left\{2\right\}=\left[\begin{array}{cc} 1.6 & 0.5 \end{array}\right]$, $C_{d}\left\{1\right\}=\left[\begin{array}{cc} 0.15 & 0.15 \end{array}\right]$, $D\left\{2\right\}=0.6$, $F\left\{2\right\}=\left[\begin{array}{c} 1\\ 0.4 \end{array}\right]$, $G\left\{2\right\}=0.5$, $H\left\{2\right\}=\left[\begin{array}{c} 0.3\\ 0.1 \end{array}\right]$, $L\left\{1\right\}=\left[\begin{array}{cc} 2 & 1 \end{array}\right]$, $L_{d}\left\{2\right\}=\left[\begin{array}{cc} 0.3 & 0.1 \end{array}\right]$, $T\left\{2\right\}=\left[1.\begin{array}{cc} 3 & 1 \end{array}\right]$, $M_{1}\left\{2\right\}=\left[\begin{array}{cc} 0.2 & 0\\ 0 & 0.2 \end{array}\right]$, $M_{2}\left\{2\right\}=\left[\begin{array}{cc} 0.2 & 0\\ 0 & 0.2 \end{array}\right]$, $N\left\{2\right\}=\left[\begin{array}{cc} 0.3 & 0\\ 0 & 0.3 \end{array}\right]$.

**Figure 2:**
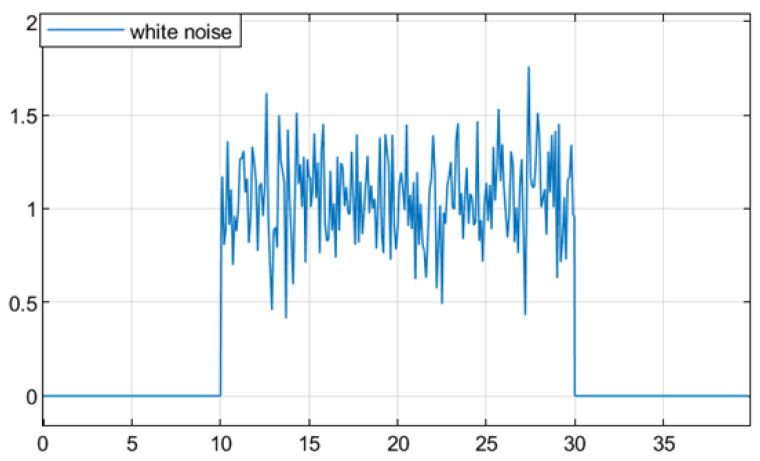
Gaussian white noise signal.

For the convenience of simulation, a white noise signal with a variance less than 0.1 and a mean value of 0.5 is chosen as the average for the system disturbance input ω(t). At time 10≤t≤30, the time-varying delay d(t) is set to satisfy 0.1≤d(t)≤0.6 and $\bar{d}\left(t\right)\leq 0.3$. Figure 2 shows the white noise signal.

In this study, the simulation experiments adopt a widely used fault expression f(t) in the control field taking into account the possible fault signal. The fault detection results generated by this fault expression are intuitive and clear, facilitating the evaluation of the effectiveness of the fault detection filter and ensuring comparability with existing research. The specific fault signal f(t) is expressed as follows:

(72) $$f\left(k\right)=\left\{\begin{array}{c} \begin{array}{cc} 5, & 10\leq k\leq 30 \end{array}\\ \begin{array}{cc} 0, & others \end{array} \end{array}\right.$$

By using Theorem 3, let $S={\left[\begin{array}{cc} 0 & 1 \end{array}\right]}^{T}$ and γ=1, it is possible to determine the parameters of the desired full-order FDF. as shown below:

The full order FDF parameter matrix of TRM:

(73) $$\begin{array}{c} A_{f}\left\{1\right\}=\left[\begin{array}{cc} -0.29 & -0.04\\ -0.04 & -0.102 \end{array}\right],B_{f}\left\{1\right\}=\left[\begin{array}{c} -0.147\\ 0.3697 \end{array}\right]\\ A_{f}\left\{2\right\}=\left[\begin{array}{cc} -0.107 & 0.072\\ 0.072 & 0.151 \end{array}\right],B_{f}\left\{2\right\}=\left[\begin{array}{c} -0.0971\\ 0.1873 \end{array}\right]\\ C_{f}\left\{1\right\}=\left[\begin{array}{cc} -0.7273 & -1.237 \end{array}\right],D_{f}\left\{1\right\}=0.0126\\ C_{f}\left\{2\right\}=\left[\begin{array}{cc} -0.941 & -0.6169 \end{array}\right],D_{f}\left\{2\right\}=0.2409 \end{array}$$

**Figure 3:**
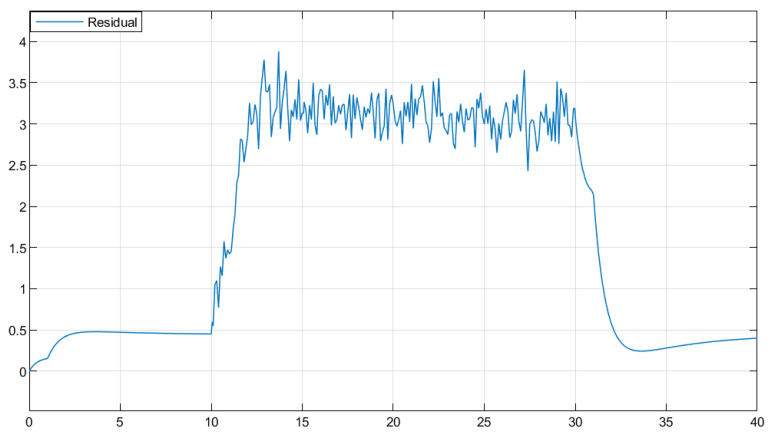
Residual Function.

**Figure 4:**
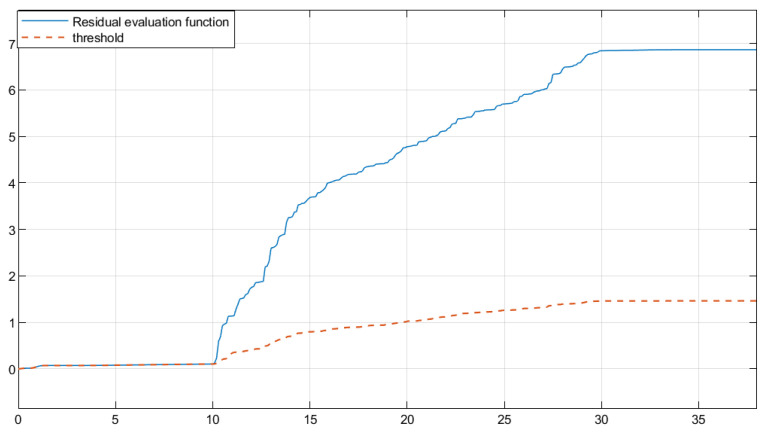
Residual evaluation function curve and threshold of full order FDF.

The residual function was obtained through simulation in Figure 3. In Figure 4, the solid line represents the residual evaluation function curve J(r(t)) when there is a fault, and the dashed line represents the residual evaluation function curve $J_{th}$ when there is no fault. The curve without fault is set as the threshold. As can be seen from Figure 3, when the time step is greater than 30 s, the fault disappears and the image tends to stabilize. The threshold is $J_{th}$ ≈ 1.452, $J\left(r\left(t\right)\right)={\left\{\int_{0}^{12}r^{T}\left(t\right)r\left(t\right)\right\}}^{1/2}=1.512>J_{th}$. This proves that the designed fault detection filter can complete fault detection within a limited time.

**Remark** **5.**  *Through this simulation analysis, it can be seen that for numerical examples that conform to this complex system, when there are time-varying delays, disturbances, uncertainties and other conditions in the system, the system can operate stably under the premise of Theorem 3. When the system fails at 10 s, the residual value increases rapidly, the residual evaluation function value quickly exceeds the reference value and gradually rises. When the fault disappears at 30 s, The residual evaluation function values began to stabilize, and the residual values also started to recover. Experiments have proved that this fault detection filter is effective for numerical examples that meet the conditions.*

**Example** **2.**  *Consider a load driven by a DC motor, where the switching of the servo DC motor is represented using a continuous-time Markov process*  $\left\{r(t),t>0\right\}$*. Based on the desired system characteristics, the inductance*  $L_{m}$ *of the servo DC motor is neglected. Let*  I(t) *denote the motor current at time*  *t,* u(t) *indicate the motor voltage at time*  *t, and*  W(t) *express the rotational speed of the motor shaft at time t. According to the basic electrical and mechanical principles governing the motor, the following equations can be derived:*

*DC servo motor model:*

(74) $$\left\{\begin{array}{c} \dot{W}\left(t\right)=-\frac{B_{i}}{J_{i}}W\left(t\right)+\frac{K_{t}}{J_{i}}I\left(t\right)+f\left(t\right)+\omega \left(t\right)\\ u\left(t\right)=K_{w}W\left(t\right)+RI\left(t\right)-\phi \left(W,t\right) \end{array}\right.$$

*where*  W(t) *is the axis velocity;*  $\dot{W}\left(t\right)$ *is the derivative of *W(t); f(t) *is a sensor malfunction;*  ϕ(W,t) *is the frictional resistance;*  u(t) *is the system input;*  $K_{w}$ *denotes the torque constant;*  *R*  *is the resistance;*  ω(t) *is the perturbation term.*

Let $W\left(t\right)=\zeta_{1}\left(t\right)$, $I\left(t\right)=\zeta_{2}\left(t\right)$, $\zeta \left(t\right)=\left[\begin{array}{c} \zeta_{1}\left(t\right)\\ \zeta_{2}\left(t\right) \end{array}\right]$, the system (72) can become:

(75) $$\left[\begin{array}{cc} 1 & 0\\ 0 & 0 \end{array}\right]\dot{\zeta }\left(t\right)=\left[\begin{array}{cc} -\frac{B_{i}}{J_{i}} & \frac{K_{t}}{J_{i}}\\ -K_{\omega } & -R \end{array}\right]\zeta \left(t\right)+\left[\begin{array}{c} 0\\ 1 \end{array}\right]\left(u(t)+\phi (W,t)\right)+\left[\begin{array}{c} 1\\ 0 \end{array}\right]f\left(t\right)+\left[\begin{array}{c} 1\\ 0 \end{array}\right]\omega \left(t\right)$$

Assuming time-varying delays d(t)=0.1+0.05sin2t, the system (73) can be transformed into:

(76) $$\begin{array}{c} \left[\begin{array}{cc} 1 & 0\\ 0 & 0 \end{array}\right]\dot{\zeta }\left(t\right)=\left[\begin{array}{cc} -\frac{B_{i}}{J_{i}} & \frac{K_{t}}{J_{i}}\\ -K_{W} & -R \end{array}\right]\zeta \left(t\right)+\left[\begin{array}{c} 0\\ 1 \end{array}\right]\left(u(t)+\phi (W,t)\right)+\left[\begin{array}{c} 1\\ 0 \end{array}\right]f\left(t\right)+\left[\begin{array}{c} 1\\ 0 \end{array}\right]\omega \left(t\right)\\ +\left[\begin{array}{cc} 0 & 0\\ 0.5 & 0 \end{array}\right]x\left(t-d\left(t\right)\right) \end{array}$$

By introducing uncertain information into system (74), we can obtain:

(77) $$\begin{array}{c} E\dot{x}\left(t\right)=\left(A_{i}+\Delta A_{i}\right))x\left(t\right)+\left(A_{di}+\Delta A_{di}\right)x\left(t-d\left(t\right)\right)+B_{i}\left(u\left(t\right)+\Phi \left(x\left(t\right),t\right)\right)\\ +F_{i}f\left(t\right)+H_{i}\omega \left(t\right) \end{array}$$

**Figure 5:**
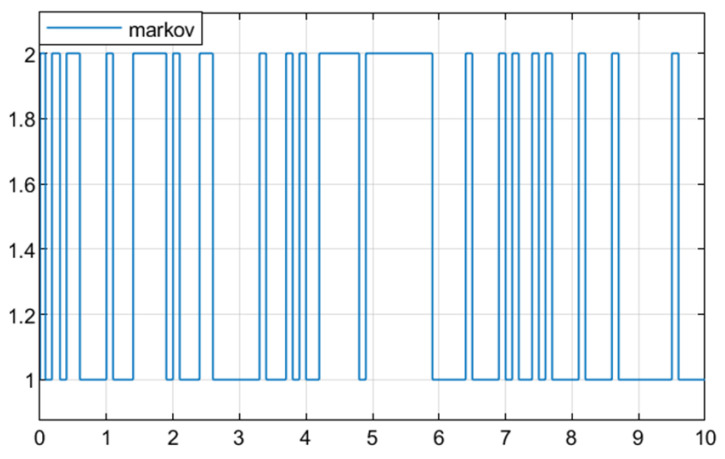
The Markov process.

Let the transfer rate be $\lambda_{ij}=\left[\begin{array}{cc} -0.8 & 0.8\\ 0.6 & -0.6 \end{array}\right]$, where Figure 5 shows the Markov process at this transfer rate.

where
When i=1:

$A\left\{1\right\}=\left[\begin{array}{cc} 1 & 0\\ 3 & 2 \end{array}\right]$, $A_{d}\left\{1\right\}=\left[\begin{array}{cc} 0 & 0\\ 0.5 & 0 \end{array}\right]$, $B\left\{1\right\}=\left[\begin{array}{c} 2\\ 1 \end{array}\right]$, $C\left\{1\right\}=\left[\begin{array}{cc} 1 & 0.6 \end{array}\right]$, $C_{d}\left\{1\right\}=\left[\begin{array}{cc} 0.1 & 0.2 \end{array}\right]$, $D\left\{1\right\}=0.3$, $F\left\{1\right\}=\left[\begin{array}{c} 0.7\\ 0.8 \end{array}\right]$, $G\left\{1\right\}=0.1$, $H\left\{1\right\}=\left[\begin{array}{c} 0.2\\ 0.1 \end{array}\right]$, $L\left\{1\right\}=\left[\begin{array}{cc} 2 & 1 \end{array}\right]$, $L_{d}\left\{1\right\}=\left[\begin{array}{cc} 0.2 & 0.1 \end{array}\right]$, $T\left\{1\right\}=\left[\begin{array}{cc} 2 & 1 \end{array}\right]$, $M_{1}\left\{1\right\}=\left[\begin{array}{cc} 0.5 & 0\\ 0 & 0.1 \end{array}\right]$, $M_{2}\left\{1\right\}=\left[\begin{array}{cc} 0.5 & 0\\ 0 & 0.6 \end{array}\right]$, $N\left\{1\right\}=\left[\begin{array}{cc} 0.5 & 0\\ 0 & 0.1 \end{array}\right]$.

When i=2:

$A\left\{2\right\}=\left[\begin{array}{cc} 3 & 0\\ 0 & 2 \end{array}\right]$, $A_{d}\left\{2\right\}=\left[\begin{array}{cc} 0 & 0\\ 0.5 & 0 \end{array}\right]$, $B\left\{2\right\}=\left[\begin{array}{c} 3\\ 1 \end{array}\right]$, $C\left\{2\right\}=\left[\begin{array}{cc} 0.6 & 0.5 \end{array}\right]$, $C_{d}\left\{2\right\}=\left[\begin{array}{cc} 0.3 & 0.5 \end{array}\right]$, $D\left\{2\right\}=0.4$, $F\left\{2\right\}=\left[\begin{array}{c} 0.4\\ 0.3 \end{array}\right]$, $G\left\{2\right\}=0.2$, $H\left\{2\right\}=\left[\begin{array}{c} 0.3\\ 0.1 \end{array}\right]$, $L\left\{2\right\}=\left[\begin{array}{cc} 1 & 0 \end{array}\right]$, $L_{d}\left\{2\right\}=\left[\begin{array}{cc} 0.3 & 0.1 \end{array}\right]$, $T\left\{2\right\}=\left[\begin{array}{cc} 3 & 1 \end{array}\right]$, $M_{1}\left\{2\right\}=\left[\begin{array}{cc} 0.2 & 0\\ 0 & 0.2 \end{array}\right]$, $M_{2}\left\{2\right\}=\left[\begin{array}{cc} 0.2 & 0\\ 0 & 0.2 \end{array}\right]$, $N\left\{2\right\}=\left[\begin{array}{cc} 0.2 & 0\\ 0 & 0.2 \end{array}\right]$.

By using Theorem 2, let $S={\left[\begin{array}{cc} 0 & 1 \end{array}\right]}^{T}$ and γ=1, it is possible to determine the parameters of the desired full-order FDF. as shown below:

(78) $$\begin{array}{c} A_{f}\left\{1\right\}=\left[\begin{array}{cc} 0.0025 & 0.1144\\ 0.1144 & -0.1112 \end{array}\right],B_{f}\left\{1\right\}=\left[\begin{array}{c} 0.082\\ -0.033 \end{array}\right]\\ A_{f}\left\{2\right\}=\left[\begin{array}{cc} 0.0033 & 0.0021\\ 0.0021 & 0.0066 \end{array}\right],B_{f}\left\{2\right\}=\left[\begin{array}{c} -0.0239\\ 0.0112 \end{array}\right]\\ C_{f}\left\{1\right\}=\left[\begin{array}{cc} -0.0436 & -0.277 \end{array}\right],D_{f}\left\{1\right\}=0.0176\\ C_{f}\left\{2\right\}=\left[\begin{array}{cc} -0.103 & -0.113 \end{array}\right],D_{f}\left\{2\right\}=0.1988 \end{array}$$

**Figure 6:**
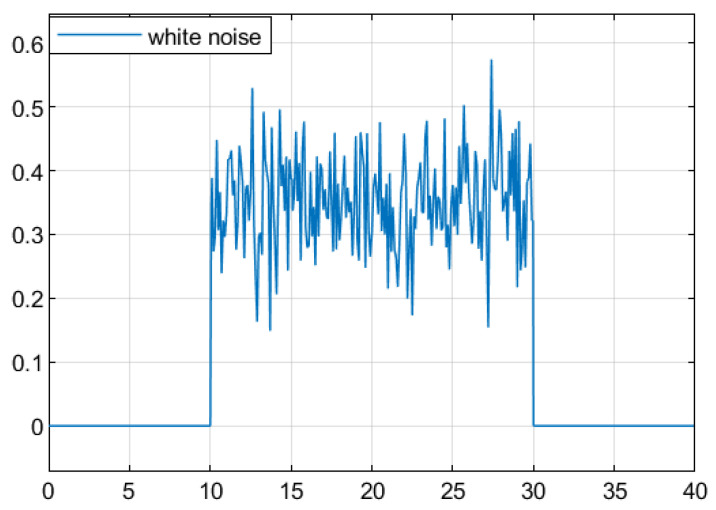
Gaussian white noise signal.

Figure 5 shows the random switching process of the DC motor system, and Figure 6 shows the randomly disturbed Gaussian white noise of the selected system.

**Figure 7:**
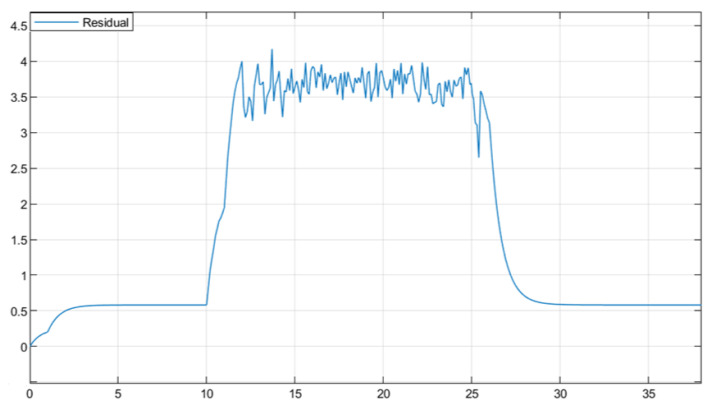
Residual Function.

**Figure 8:**
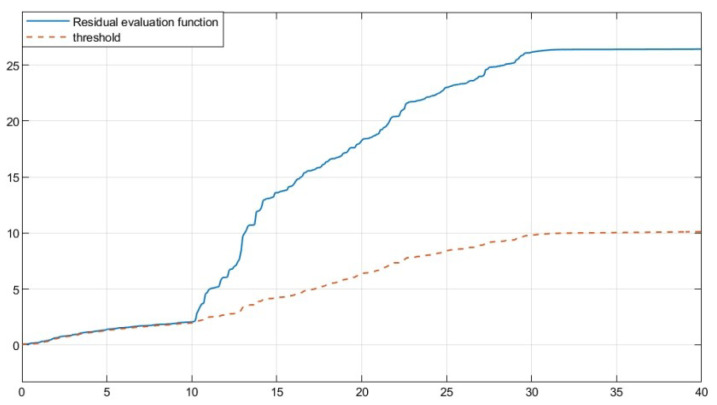
Residual evaluation function curve and threshold of full order FDF.

Based on the obtained parameters, simulation analysis was conducted through Simulink, and the residual function curve could be obtained, as shown in Figure 7. In Figure 8, the solid line represents the residual evaluation function curve J(r(t)) when there is a fault, and the dashed line represents the residual evaluation function curve $J_{th}$ when there is no fault. The curve without a fault is set as the threshold. As can be seen from Figure 8, when the time step is greater than 30, the fault disappears and the image tends to stabilize. The threshold is $J_{th}$ ≈ 10.136, $J\left(r\left(t\right)\right)={\left\{\int_{0}^{13}r^{T}\left(t\right)r\left(t\right)\right\}}^{1/2}=10.375>J_{th}$. It can be concluded that when t=13 s, the filter can complete the expected fault detection, thereby proving the effectiveness of the fault detection filter.

**Remark** **6.**  *Through the simulation analysis of this DC motor system, it can be seen that when there are time-varying delays, disturbances, uncertainties and other conditions in the system, the system can also quickly detect faults under the conditions of Theorem 3, and the residual evaluation function can also tend to stabilize when the fault disappears. The simulation analysis results show that this fault detection filter is effective for fault detection in DC motor systems.*

**Example** **3.**  *Considering the linearized model from the F-404 aircraft engine system [35], the system state matrix is:*

(79) $$A=\left[\begin{array}{ccc} -1.46 & 0 & 2.428\\ 0.1643+0.5* & -0.4+* & -0.3788\\ 0.3107 & 0 & -2.23 \end{array}\right]$$

**Figure 9:**
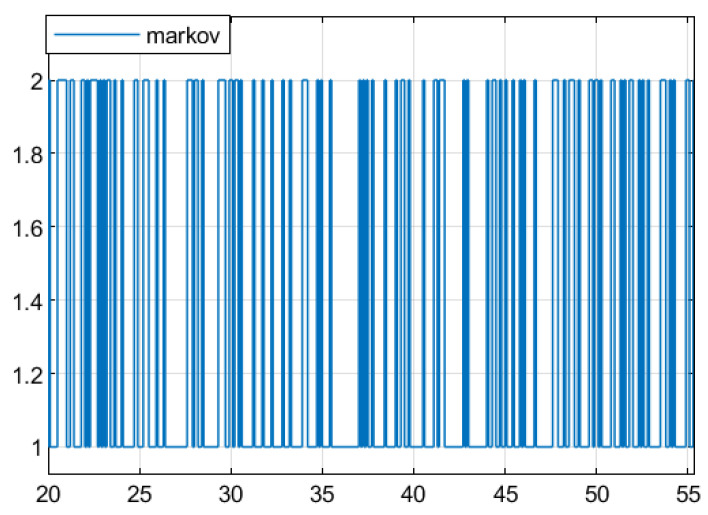
The Markov process.

*where ∗ is an uncertain model parameter. Suppose ∗ is affected by a Markov process*  r(t) *with two modes, and its transfer rate is set as*  $\lambda_{11}=-3$, $\lambda_{12}=3$, $\lambda_{21}=4$, $\lambda_{22}=-4$*. When*  r(t)=1*, the uncertainty ∗ is set to −1; And when*  r(t)=2*, it is set to −2. Figure 9 shows the Markov jump function. Based on this setting, the system matrix*  *A*  *is:*

$A_{1}=\left[\begin{array}{ccc} -1.46 & 0 & 2.428\\ -0.3357 & -1.4 & -0.3788\\ 0.3107 & 0 & -2.23 \end{array}\right]$, $A_{2}=\left[\begin{array}{ccc} -1.46 & 0 & 2.428\\ -0.8357 & -2.4 & -0.3788\\ 0.3107 & 0 & -2.23 \end{array}\right]$.

*Other system parameters are:*

*When*  r(t)=1:

$B_{1}=\left[\begin{array}{cc} 0.11 & 0.1\\ 0.2 & -2\\ 0.15 & -0.1 \end{array}\right]$, $C_{1}=\left[\begin{array}{ccc} -0.1 & 0 & 1\\ 0.15 & -2 & -0.1\\ 0.1 & 0.2 & 0.1 \end{array}\right]$, $F_{1}=\left[\begin{array}{c} 0.11\\ 0.2\\ 0.15 \end{array}\right]$, $D_{1}=\left[\begin{array}{c} 0.1\\ 0.2\\ 0.3 \end{array}\right]$, $G_{1}=\left[\begin{array}{c} 0.1\\ 0.1\\ 0.1 \end{array}\right]$, $H_{1}=\left[\begin{array}{c} 0.5\\ 0.1\\ 1.3 \end{array}\right]$.

*When*  r(t)=2:

$B_{2}=\left[\begin{array}{cc} 0.1 & 0.1\\ 0.15 & -2\\ 0.1 & -0.1 \end{array}\right]$, $C_{2}=\left[\begin{array}{ccc} -0.1 & 0 & 1\\ 0.15 & -2 & -0.5\\ 0.1 & 0.2 & 0.1 \end{array}\right]$, $F_{2}=\left[\begin{array}{c} 0.1\\ 0.15\\ 0.1 \end{array}\right]$, $D_{2}=\left[\begin{array}{c} 0.1\\ 0.15\\ 0.2 \end{array}\right]$, $G_{2}=\left[\begin{array}{c} 0.1\\ 0.1\\ 0.1 \end{array}\right]$, $H_{1}=\left[\begin{array}{c} 0.5\\ 0.1\\ 2.3 \end{array}\right]$.

In this study, the simulation experiment adopted the fault expression f(t), which is widely used in the control field, as the fault signal. The specific fault signal f(t) expression is as follows:

(80) $$f\left(t\right)=\left\{\begin{array}{c} 2,20\leq t\leq 70\\ 0,\mathrm{others} \end{array}\right.$$

For the convenience of simulation, a white noise signal with a variance less than 0.01 and a mean value of 0.1 is selected as the system interference input ω(t). When 20≤t≤70, set the numerical range of the time-varying delay d(t) to satisfy d(t)≤0.3 and $\dot{d}\left(t\right)\leq 0.2$.

Based on the above conditions, the expected parameters of the fault detection filter can be obtained as follows:

$\begin{array}{c} A_{f}\left\{1\right\}=\left[\begin{array}{ccc} -1.146 & -0.182 & -0.036\\ -0.117 & -2.182 & 0.968\\ 2.594 & -2.033 & -0.781 \end{array}\right],A_{f}\left\{2\right\}=\left[\begin{array}{ccc} -2.107 & 0.172 & 0.336\\ -0.172 & 1.151 & 0.667\\ 1.852 & -2.547 & 0.538 \end{array}\right],\\ C_{f}\left\{1\right\}=\left[\begin{array}{ccc} -0.254 & -1.277 & 1.233\\ 0.574 & -0.447 & 0.457\\ 1.437 & 1.247 & -0.543 \end{array}\right],C_{f}\left\{2\right\}=\left[\begin{array}{ccc} -1.547 & -0.957 & 0.345\\ 0.454 & -0.785 & 0.138\\ -0.112 & -1.244 & -0.475 \end{array}\right], \end{array}$, $B_{f}\left\{1\right\}=\left[\begin{array}{c} -1.047\\ 0.634\\ 0.47 \end{array}\right],B_{f}\left\{2\right\}=\left[\begin{array}{c} -0.971\\ 0.187\\ 0.852 \end{array}\right],D_{f}\left\{1\right\}=\left[\begin{array}{c} 0.147\\ 0.334\\ 0.792 \end{array}\right],D_{f}\left\{2\right\}=\left[\begin{array}{c} -0.647\\ 0.549\\ 0.247 \end{array}\right]$.

**Figure 10:**
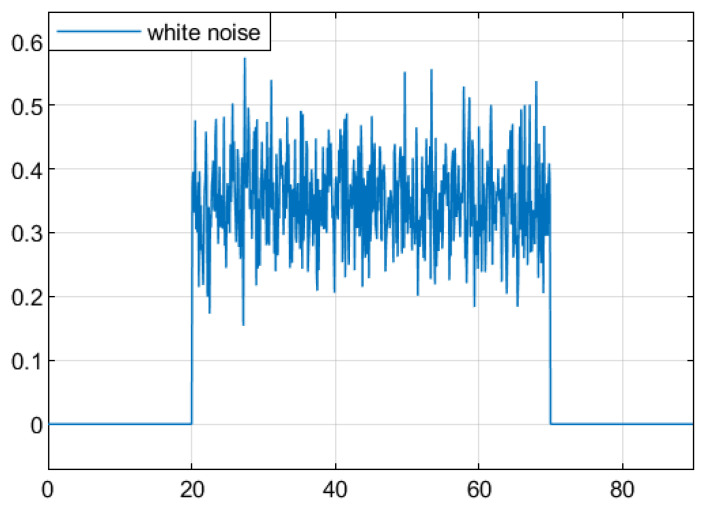
Gaussian white noise signal.

Figure 9 shows the random switching process of the engine system of the F-404 aircraft, and Figure 10 shows the randomly disturbed Gaussian white noise of the selected system.

**Figure 11:**
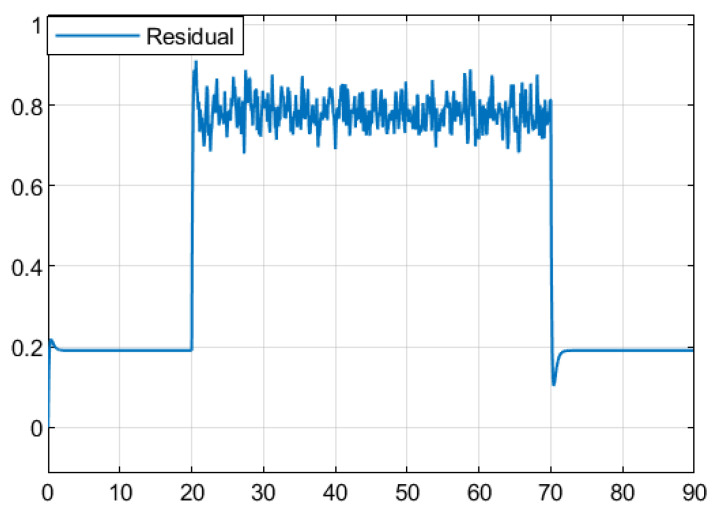
Residual Function.

**Figure 12:**
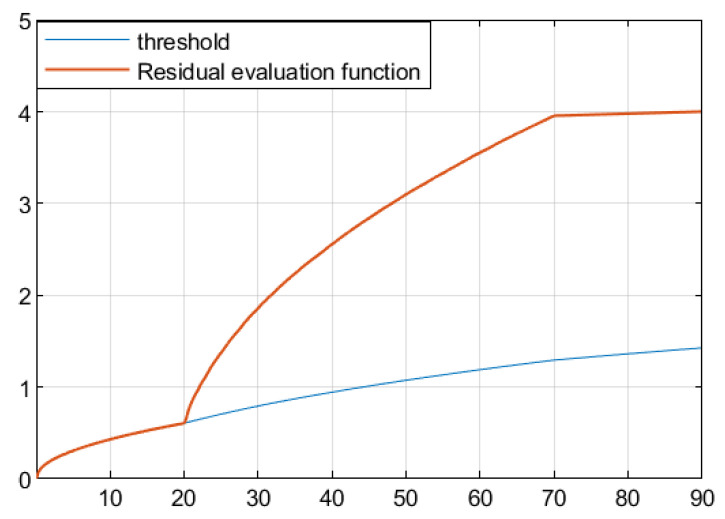
Residual evaluation function curve and threshold of full order FDF.

Based on the obtained parameters, simulation analysis was conducted through Simulink, and the residual function curve could be obtained, as shown in Figure 11. Figure 12 shows the residual evaluation function curve J(r(t)) when there is a fault, and the dotted line represents the residual evaluation function curve $J_{th}$ when there is no fault. The curve without fault is set as the threshold. According to Figure 11, when the time step is longer than 70 s, the fault disappears and the image tends to stabilize. Based on the calculated thresholds $J_{th}$ ≈ 1.446 and $J\left(r\left(t\right)\right)={\left\{\int_{0}^{23}r^{T}\left(t\right)r\left(t\right)\right\}}^{1/2}=1.561>J_{th}$, it is expected that the fault can be detected at 23 s, thereby proving the effectiveness of this fault detection filter.

**Remark** **7.**  *Through this simulation analysis of the F-404 aircraft engine system, it can be seen that when the aircraft runs for 20 s, the system malfunctions, the residual value increases, the residual evaluation function value exceeds the threshold baseline and gradually rises. When the fault disappears at 70 s, the system begins to stabilize, the residual value also starts to stabilize, and the residual evaluation function value also begins to tend to stabilize. The simulation analysis results show that this fault detection filter is effective for the fault detection of the F-404 aircraft engine system.*

## 8. Conclusions

This paper mainly studies Sensor Fault Detection and Reliable Control of Singular Stochastic Systems with Time-Varying Delays. To handle unknown nonlinear problems, based on the equivalent control principle, an integral sliding mode controller was designed to convert the unknown nonlinear influence into system input. Then, a fault detection filter adapted to this system was designed, thereby obtaining this UNSSUAFRS model. Furthermore, in order to reduce the conservatism of the system, a new summation inequality method and Wirtinger inequality are adopted to handle the time-delay problem. By using weak infinitesimal generators, the required Lyapunov-Krasovskii functional is designed. With the aid of the Lyapunov principle and $H_{\infty }$ performance analysis method, the sufficient conditions that satisfy the random admissibility of this UNSSUAFRS are derived. Finally, with the help of the designed residual evaluation function and threshold, numerical examples, DC servo motor instances and the F-404 aircraft engine system were used for simulation analysis to verify the effectiveness and practicability of this fault detection filter.

## Data Availability

The datasets generated during and/or analyzed during the current study are available from the corresponding author on reasonable request.
